# Supplementary figures and images for: Age-related accumulation of de novo mitochondrial mutations in mammalian oocytes and somatic tissues
Source: PLoS Biol. 2020 Jul 15;18(7):e3000745. doi: 10.1371/journal.pbio.3000745 (PMC7363077; doi:10.1371/journal.pbio.3000745)

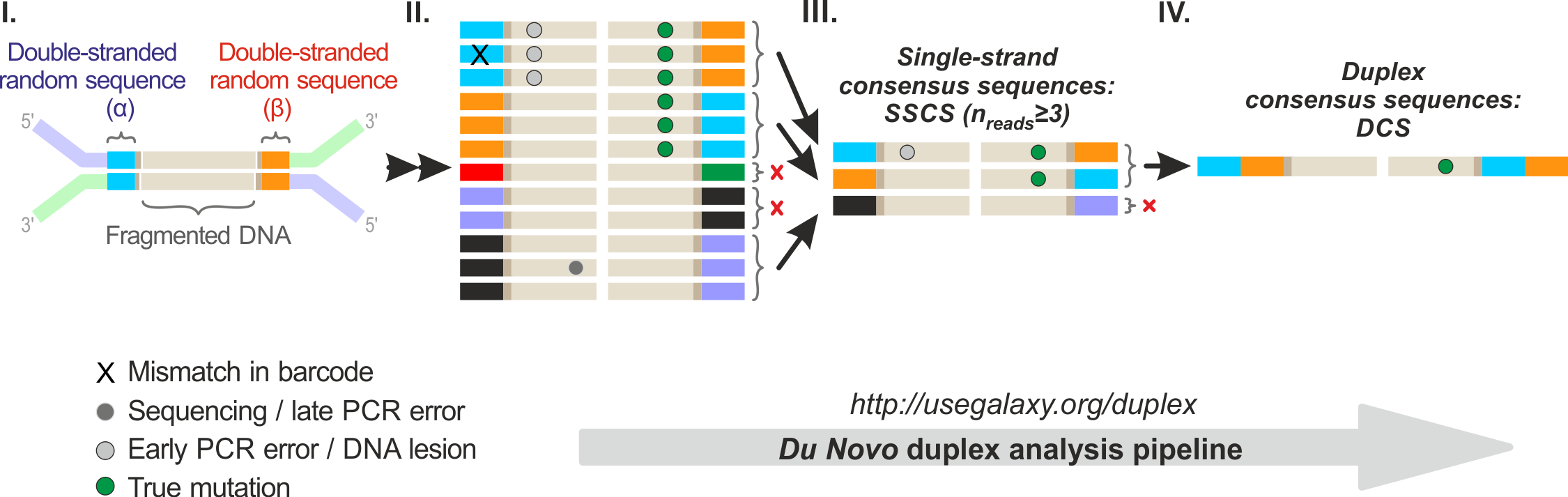

Supplement: S1 Fig — With duplex sequencing, double-stranded adapters containing a random 12-nt sequence are generated for Illumina sequencing and ligated to the double-stranded, fragmented DNA of interest. Each molecule ends up with a different combination of random sequences on both ends. Adapter-ligated molecules are amplified and sequenced, producing several sequenced reads per initial DNA strand. Reads are aligned according to their random sequences, and consensus reads are formed, first for the single strands independently, SSCS, and followed by the formation of a consensus of the two strands of a DNA duplex, DCS. The duplex sequencing principle is described in detail by Schmitt and colleagues (Fig 1 in [46]). The principle of Du Novo reference-free consensus formation with barcode error correction [51,52] is shown here. Without aligning the paired-end sequencing reads to a reference sequence, reads are directly aligned according to their random tag sequences, allowing for up to three mismatches. Mismatches are corrected, and consensus sequences are formed as described above, requiring at least three paired-end reads for the formation of a SSCS and both SSCSs for the formation of a DCS. DCS, duplex consensus sequence; SSCS, single-strand consensus sequence. (TIF) [file pbio.3000745.s001.tif]

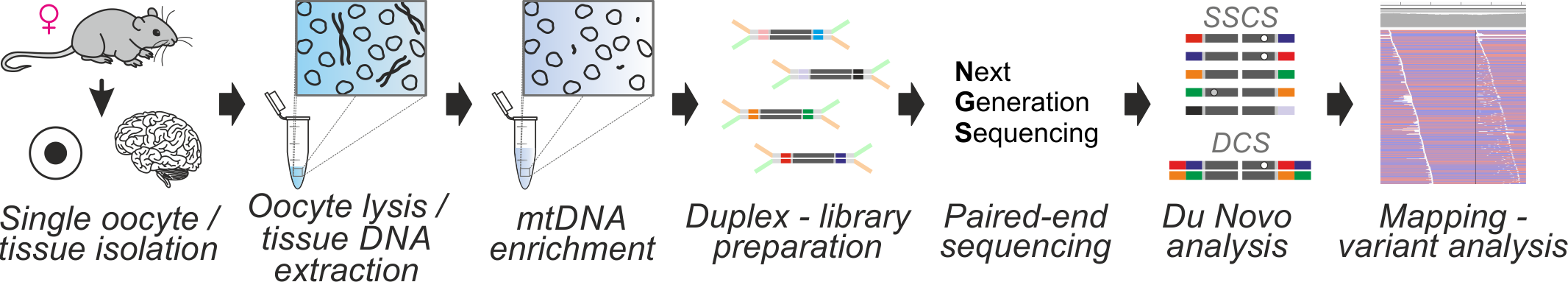

Supplement: S2 Fig — Total DNA was first extracted from somatic tissues and used as input for enzymatic mtDNA enrichment. Single oocytes or oocyte pools were lysed and directly used for the enrichment step. Duplex sequencing libraries were prepared and sequenced using 250-nt paired-end reads. Consensus formation was performed on Galaxy [95] using the Du Novo pipeline [51,52], and consensuses were mapped to the mouse mtDNA reference sequence (NC_005089.1) and further analyzed for variants. mtDNA, mitochondrial DNA. (TIF) [file pbio.3000745.s002.tif]

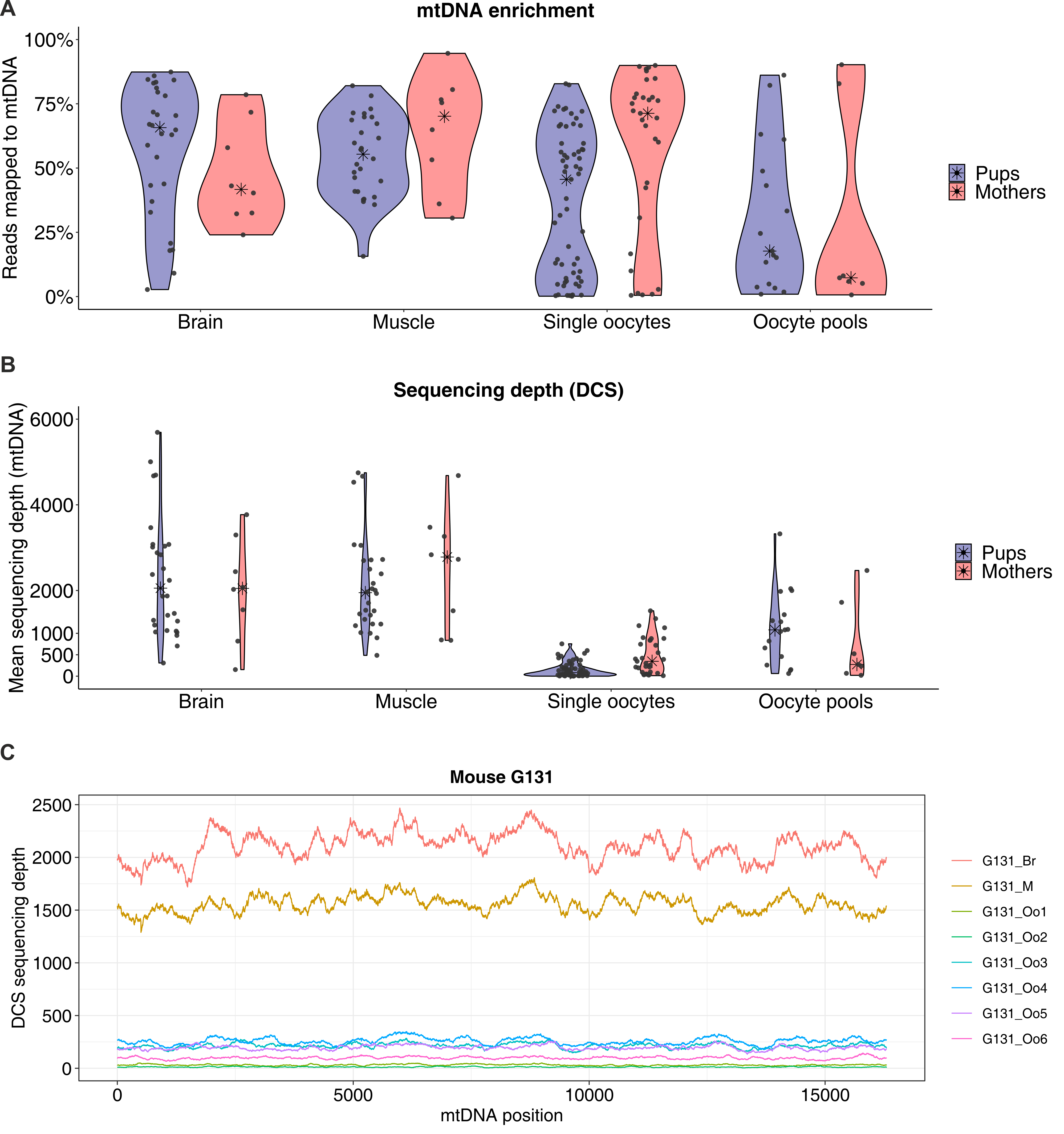

Supplement: S3 Fig — (A) The efficiency of mtDNA enrichment shows a narrower distribution in somatic tissues compared with oocytes. Asterisks indicate the median of the distribution. Larger amounts of medium in which oocytes were stored reduce enrichment efficiency, resulting in oocytes with basically no, or very poor, enrichment for samples with a large amount of medium. (B) Mean DCS sequencing depth for mtDNA. A minimum mean mtDNA sequencing depth of 500× was targeted for DCS in somatic tissues (this value in reality strongly depended on the efficiency of mtDNA enrichment and is difficult to precisely estimate before tag family amplification during duplex library preparation). For single oocytes, all molecules obtained during library preparation were used as input for tag family PCR. For samples with poor enrichment only one-fourth to one-half of the library was used because the majority of the DNA represented nuclear DNA, resulting in samples with lower mtDNA sequencing depth. Asterisks indicate the median of the distribution. (C) The distribution of DCS sequencing depth across mtDNA for a typical sample (samples from mouse G131 are shown as an example). The raw data for the information depicted in this figure are available at https://github.com/makovalab-psu/mouse-duplexSeq. DCS, duplex consensus sequence; mtDNA, mitochondrial DNA. (TIF) [file pbio.3000745.s003.tif]

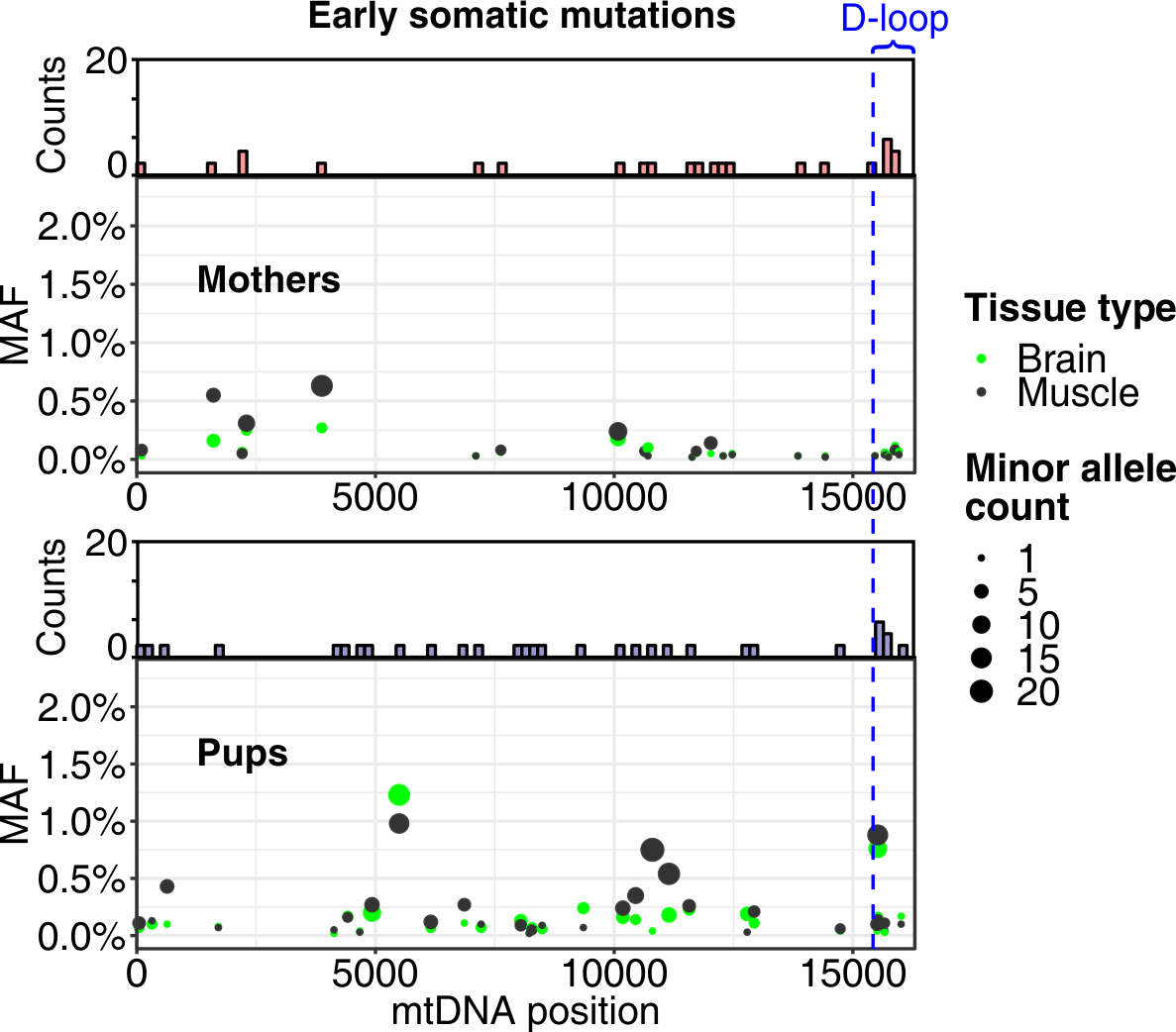

Supplement: S4 Fig — Early somatic mutations (observed in both somatic tissues). Only one of them reached MAF >1% (in brain of pup G132p1). The raw data for the information depicted in this figure are available at https://github.com/makovalab-psu/mouse-duplexSeq. MAF, minor allele frequency. (TIF) [file pbio.3000745.s004.tif]

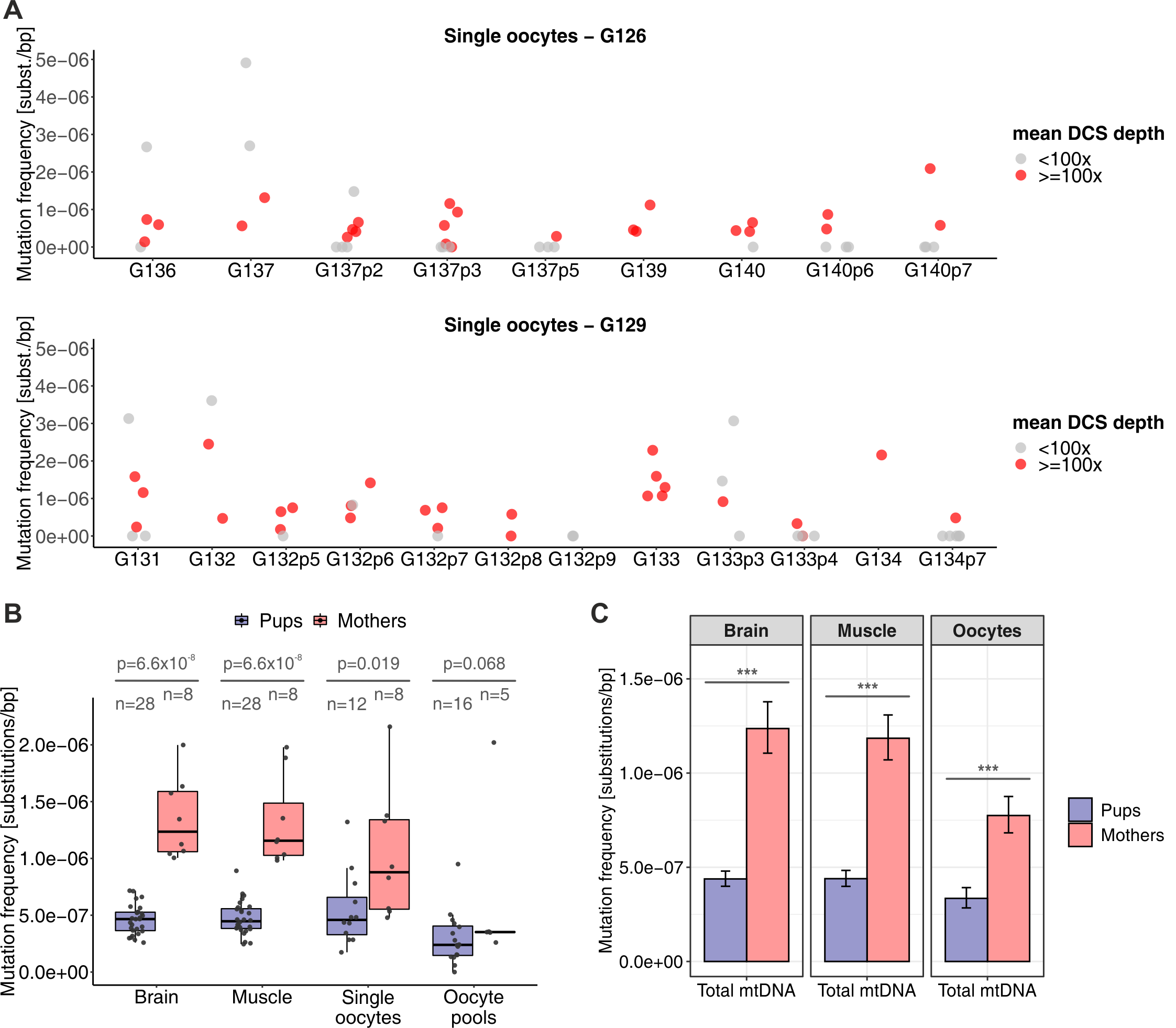

Supplement: S5 Fig — (A) Nucleotide substitution frequencies measured in individual oocytes of mothers and pups in the mouse pedigrees G126 and G129. Gray dots indicate a mean DCS sequencing depth <100×. Because of the low sequencing depth, mutation frequencies might be biased toward extremely low or high values (depending on the absence or presence of a mutation). (B) Mutation frequencies measured in brain, muscles, single oocytes, and oocyte pools of mothers and pups, shown at a per-individual level. Only samples sequenced at a depth of at least 100× were included (all somatic tissues; 51 of 92 single oocytes; 21 of 24 oocyte pools) to ensure accurate mutation frequency measures. For mutation frequency computation in single oocytes, the numbers of mutations and sequenced nucleotides were combined across all oocytes measured for the same mouse. Permutation test p-values are indicated (one-sided test based on medians; 100,000,000 permutations; corrected for multiple testing). (C) Mutation frequencies in the total mtDNA measured in brain, muscle, and oocytes (single oocytes and oocyte pools combined) of mothers and pups aggregated for all individuals of an age group. Difference between pups and mothers in each category was tested using Fisher’s exact test; *p < 0.05, **p < 0.01, ***p < 0.001. The raw data for the information depicted in this figure are available at https://github.com/makovalab-psu/mouse-duplexSeq. DCS, duplex consensus sequence; mtDNA, mitochondrial DNA. (TIF) [file pbio.3000745.s005.tif]

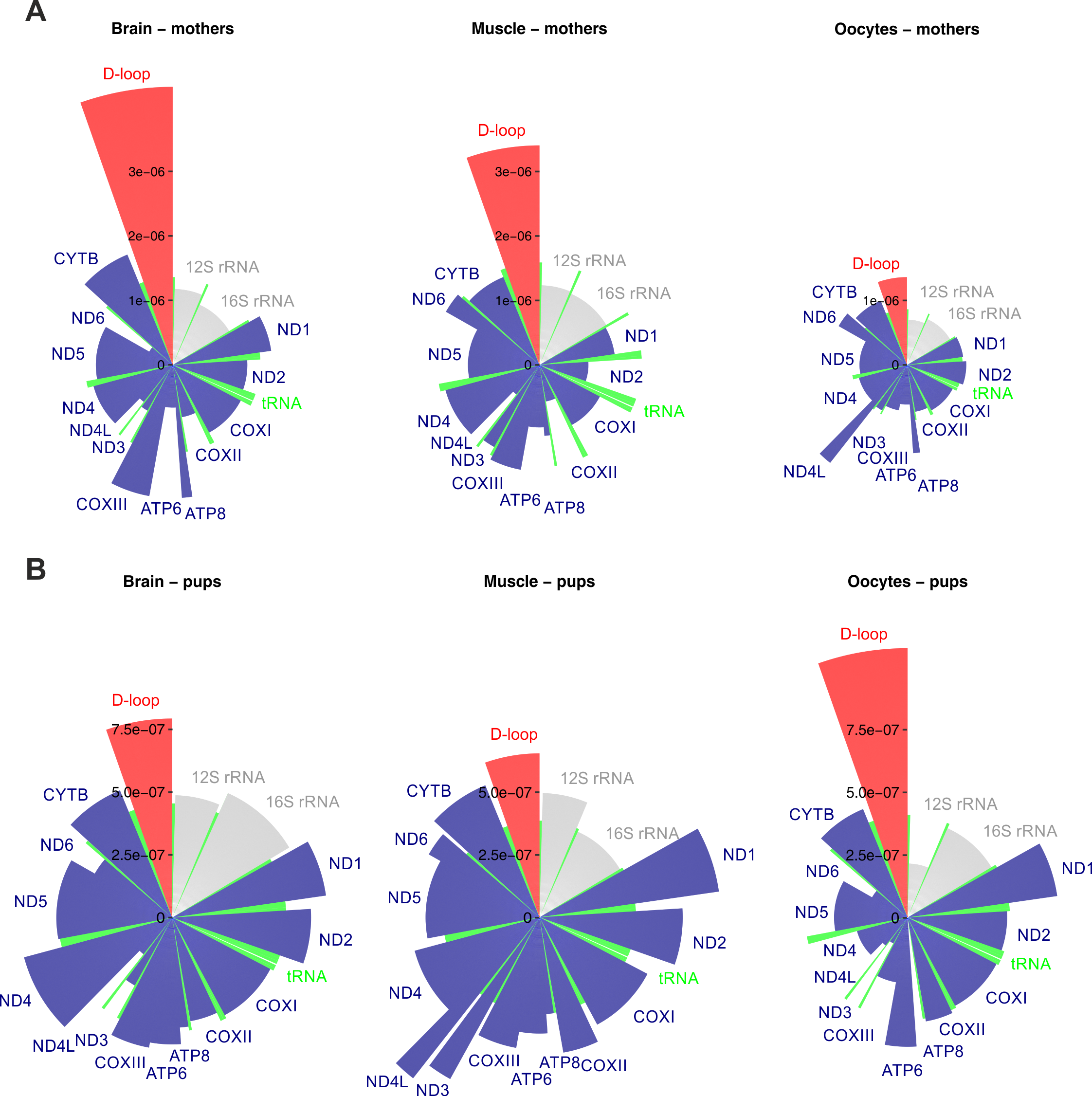

Supplement: S6 Fig — Mutation frequencies are shown for (A) mothers and (B) pups for the different regions along the mtDNA. Mutations frequencies in the tRNA coding regions (green) were aggregated. The raw data for the information depicted in this figure are available at https://github.com/makovalab-psu/mouse-duplexSeq. mtDNA, mitochondrial DNA. (TIF) [file pbio.3000745.s006.tif]

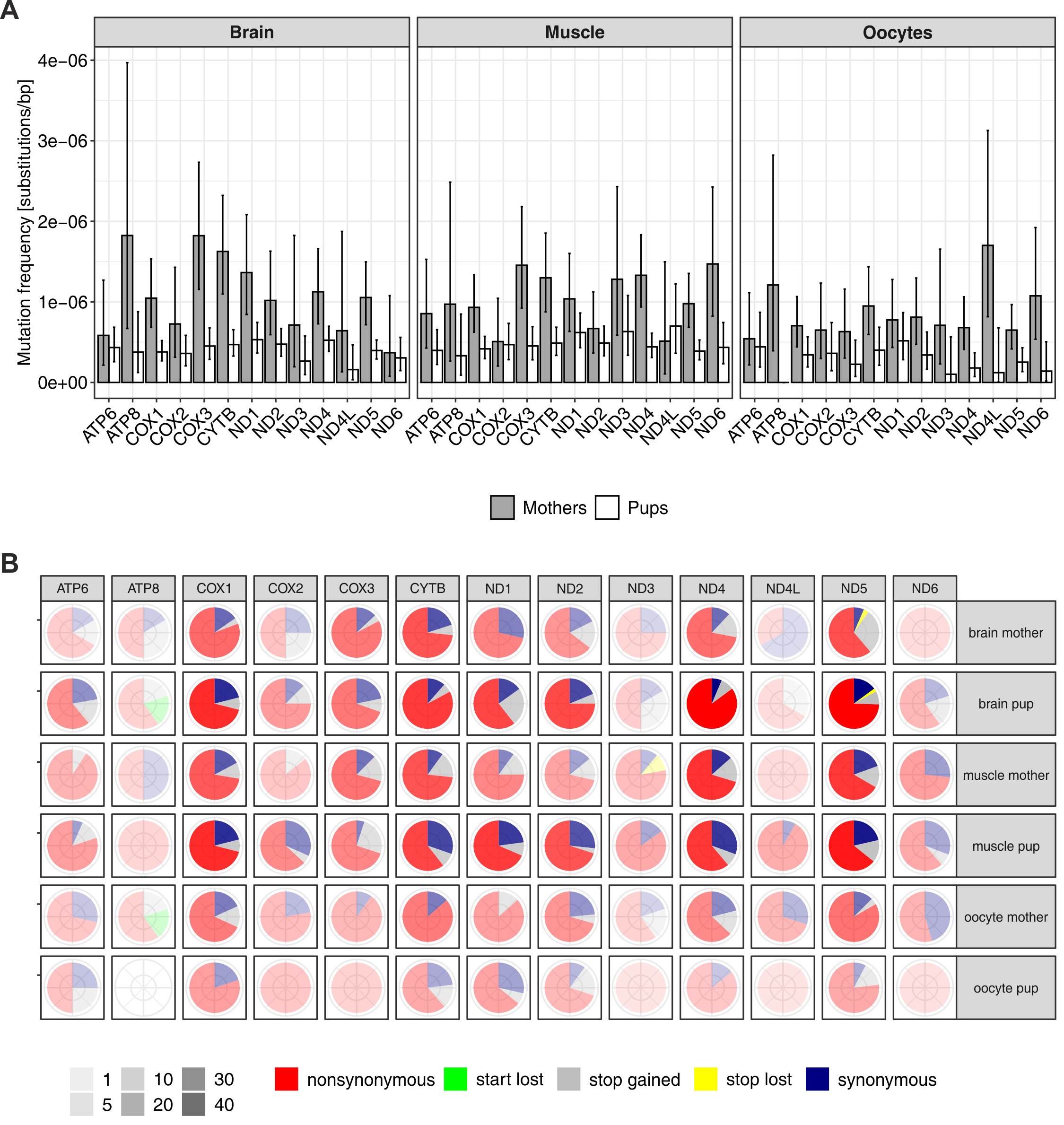

Supplement: S7 Fig — (A) Mutation frequencies within the different protein coding genes in brain, muscle, and oocytes of mothers and pup. (B) Distribution of nonsynonymous mutations (red), lost start codons (green), gained stop codons (gray), lost stop codons (yellow), and synonymous mutations (blue) within the different genes. The level of transparency represents the total number of mutations found in a gene, ranging from 1 to 47. The raw data for the information depicted in this figure are available at https://github.com/makovalab-psu/mouse-duplexSeq. (TIF) [file pbio.3000745.s007.tif]

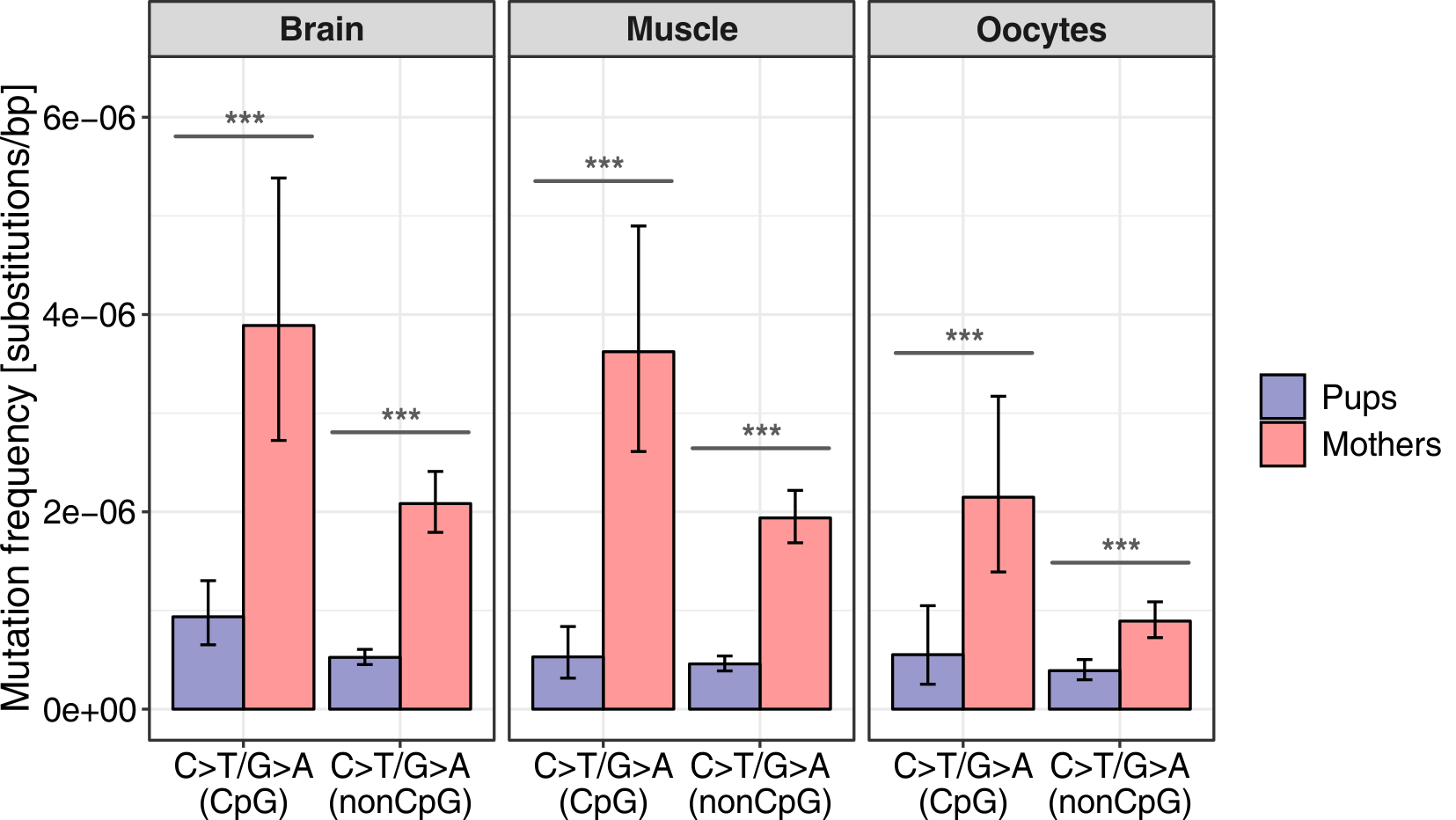

Supplement: S8 Fig — Significance of differences between mutation frequencies in mothers and pups was tested using Fisher’s exact test; * p < 0.05, ** p < 0.01, *** p<0.001; corrected for multiple testing. The raw data for the information depicted in this figure are available at https://github.com/makovalab-psu/mouse-duplexSeq. (TIF) [file pbio.3000745.s008.tif]

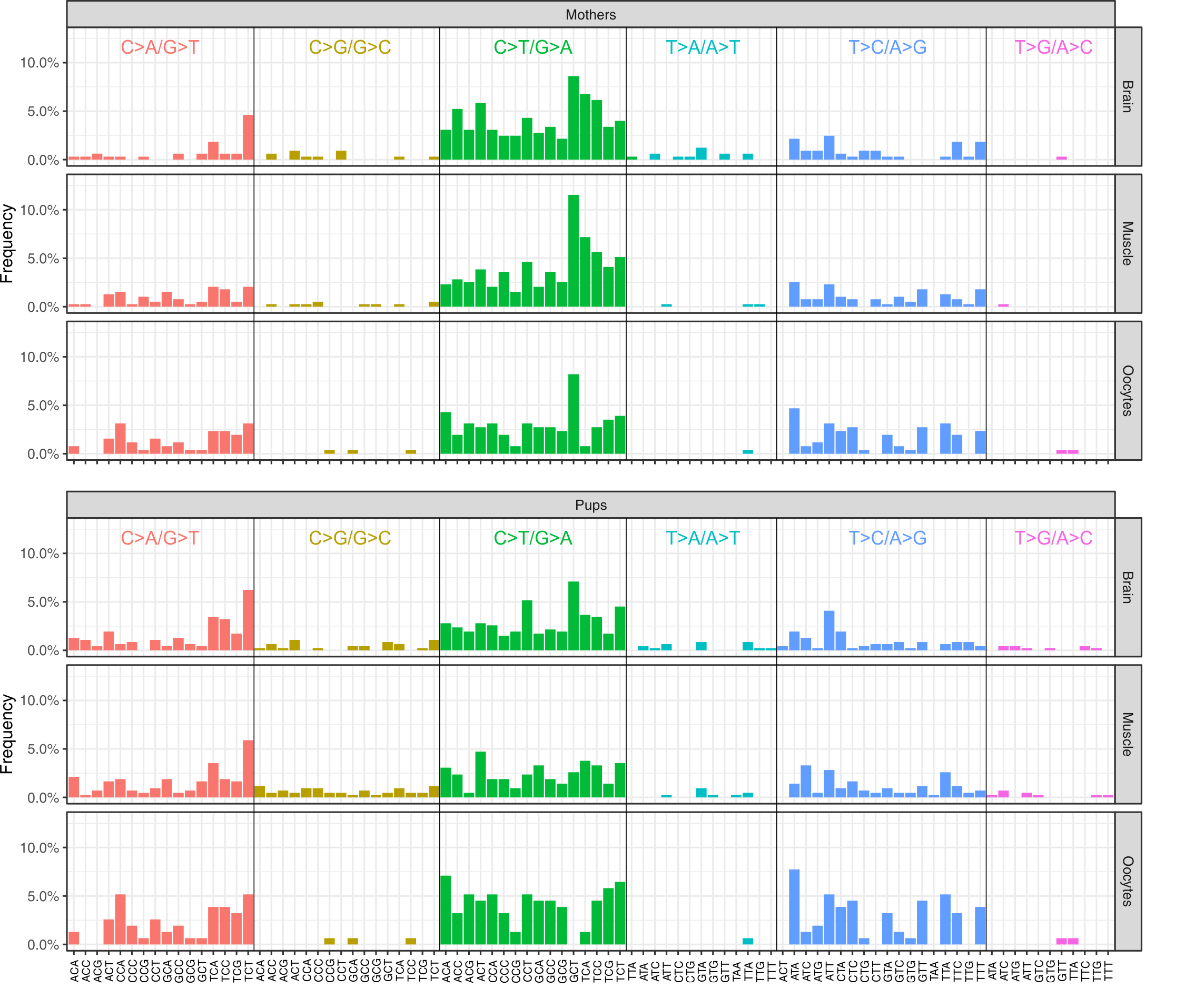

Supplement: S9 Fig — The trinucleotide context is shown for mutations in brain, muscle, and oocytes in mothers and pups, respectively. The nucleotide context (e.g., ACA) is only listed for one strand, but it represents the context on both strands (e.g., ACA for C>A mutations on one strand, and TGT for G>T mutations on the second strand). No significant differences in the trinucleotide context of mutations between mothers and pups was observed for brain, muscle, and oocytes (p = 1, p = 1, and p = 1, respectively; Pearson’s chi-squared test of independence with Monte Carlo simulations). The raw data for the information depicted in this figure are available at https://github.com/makovalab-psu/mouse-duplexSeq. (TIF) [file pbio.3000745.s009.tif]

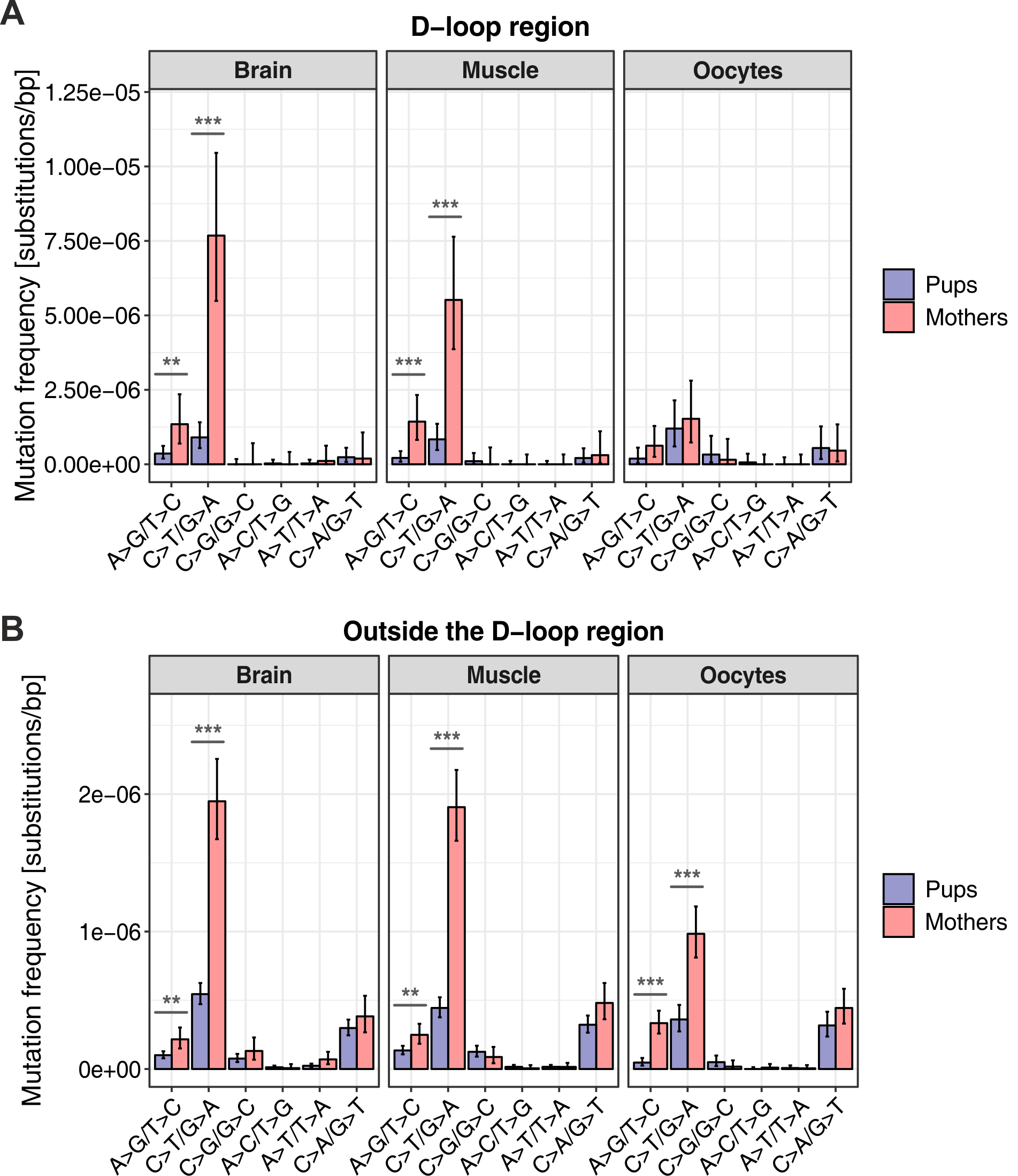

Supplement: S10 Fig — (A) Frequencies of different mutation types in the D-loop in pups and mothers. (B) Frequencies of different mutation types outside the D-loop in pups and mothers. Significance of differences between mothers and pups was tested using Fisher’s exact test; *p < 0.05, **p < 0.01, ***p < 0.001; corrected for multiple testing. The raw data for the information depicted in this figure are available at https://github.com/makovalab-psu/mouse-duplexSeq. (TIF) [file pbio.3000745.s010.tif]

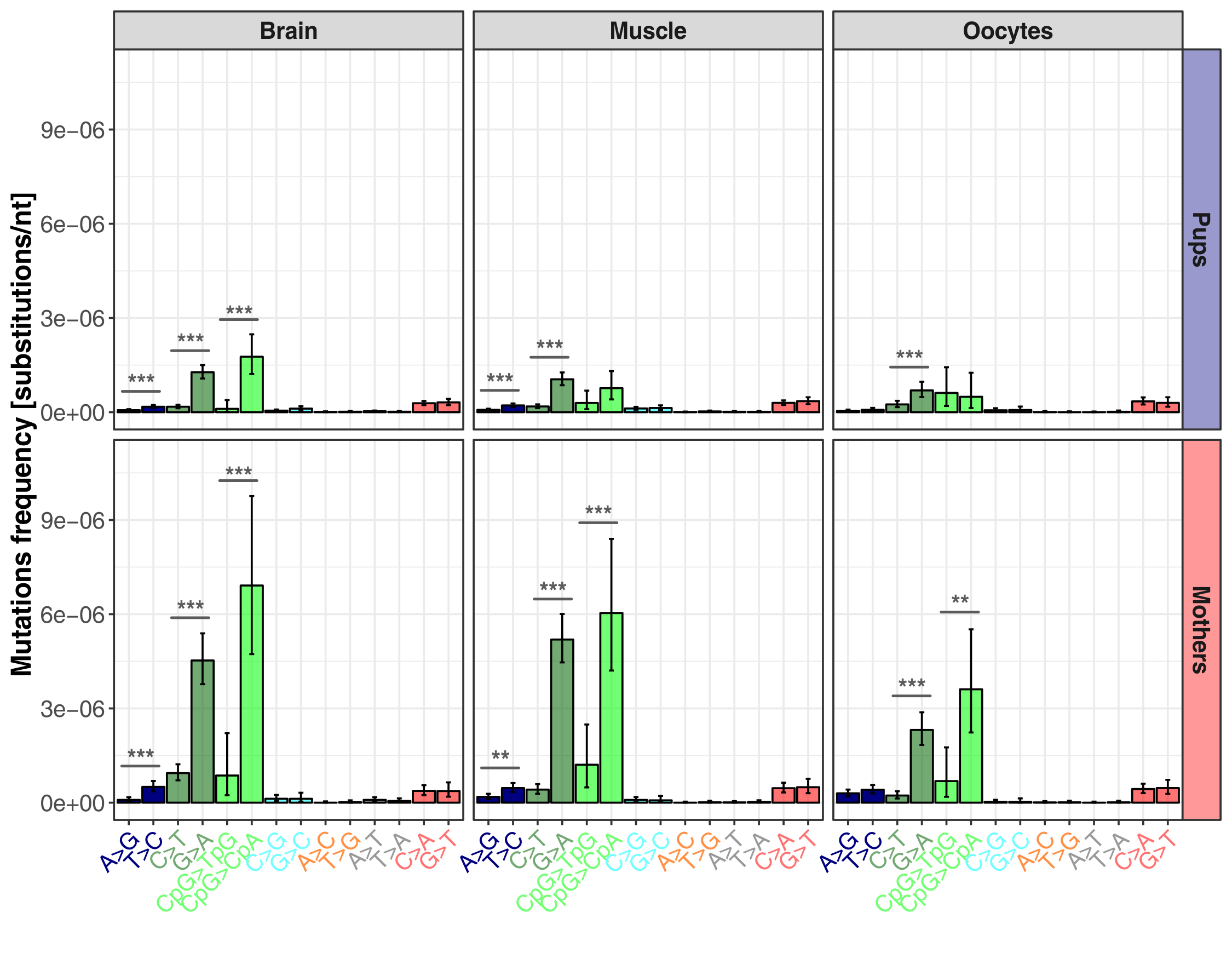

Supplement: S11 Fig — We observe an asymmetric distribution of different mutation types between the L-strand (containing more cytosines than guanines) and H-strand of mtDNA, as shown previously for human brain [28]. The substitution type is shown relative to the reference sequence (representing L-strand) of mtDNA. Significance of differences between mutations on different strands was tested using Fisher’s exact test; *p < 0.05, **p < 0.01, ***p < 0.001; corrected for multiple testing. The raw data for the information depicted in this figure are available at https://github.com/makovalab-psu/mouse-duplexSeq. H-strand, heavy strand; L-strand, light strand; mtDNA, mitochondrial DNA. (TIF) [file pbio.3000745.s011.tif]

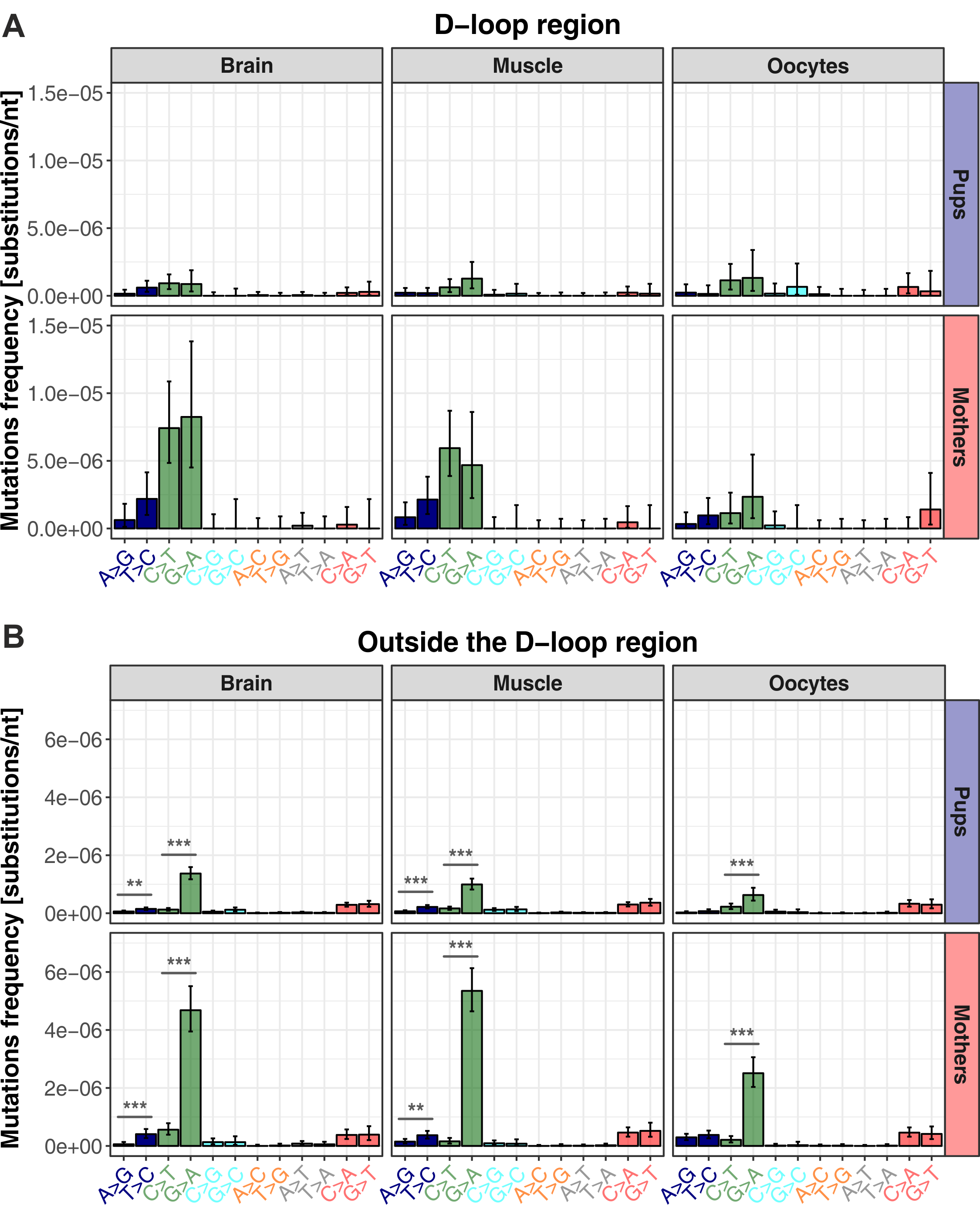

Supplement: S12 Fig — (A) Mutation frequencies of different mutation types in the D-loop in brain, muscle, and oocytes. (B) Mutation frequencies of different mutation types outside the D-loop in brain, muscle, and oocytes. Significance of differences between different strands was tested using Fisher’s exact test; *p < 0.05, **p < 0.01, ***p < 0.001; corrected for multiple testing. The raw data for the information depicted in this figure are available at https://github.com/makovalab-psu/mouse-duplexSeq. mtDNA, mitochondrial DNA. (TIF) [file pbio.3000745.s012.tif]

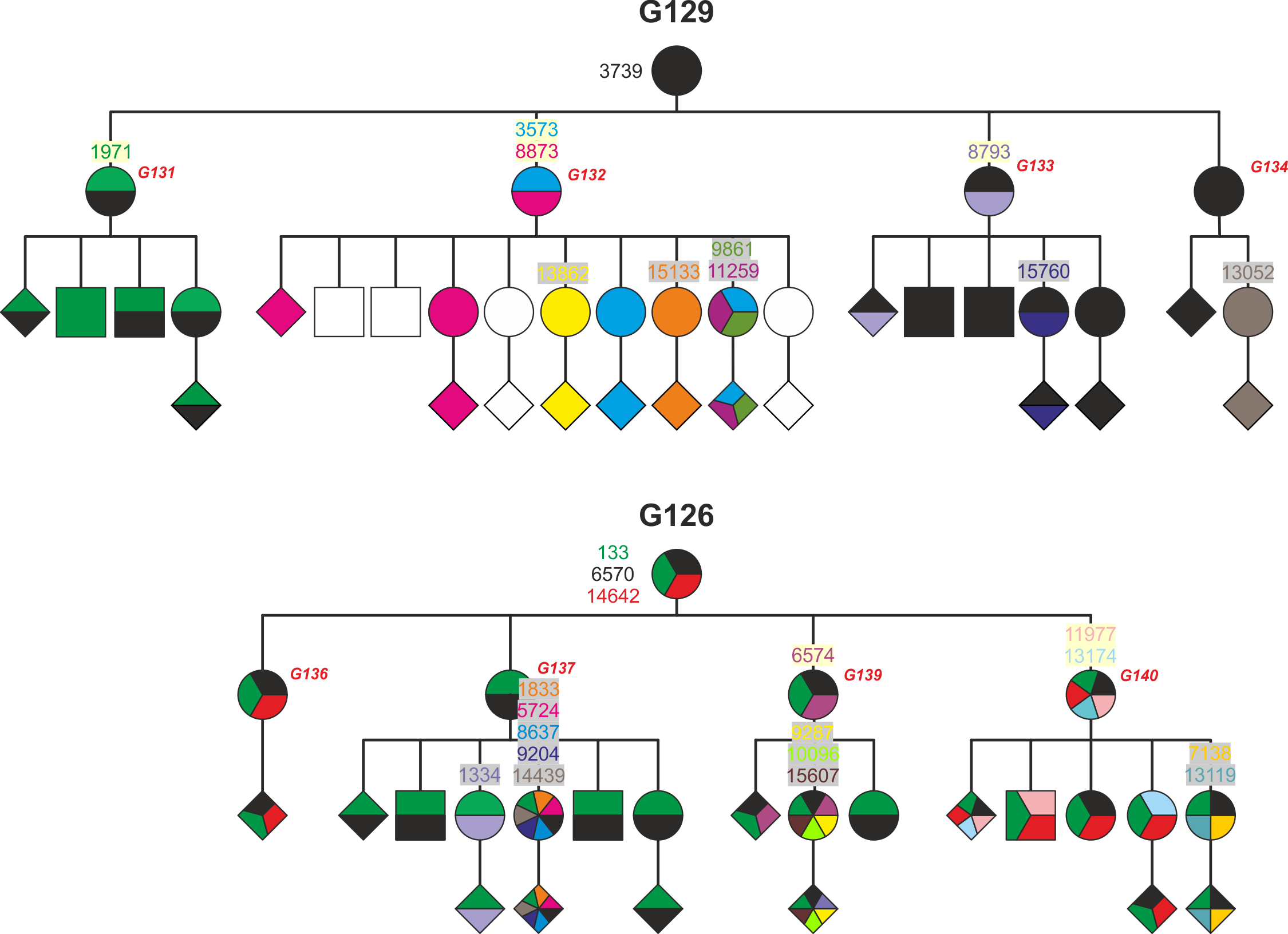

Supplement: S13 Fig — Red numbers in italics indicate the IDs of the mothers. Circles indicate females, squares indicate males, and diamonds indicate oocytes (all single oocytes and oocyte pools were considered together). Different color fillings show the presence of different heteroplasmic sites within individuals (or all oocytes). The position of a heteroplasmic site is shown in the corresponding color when first observed within a pedigree. Positions are highlighted in yellow when first observed in mothers and in gray when first observed in pups. ID, identifier. (TIF) [file pbio.3000745.s013.tif]

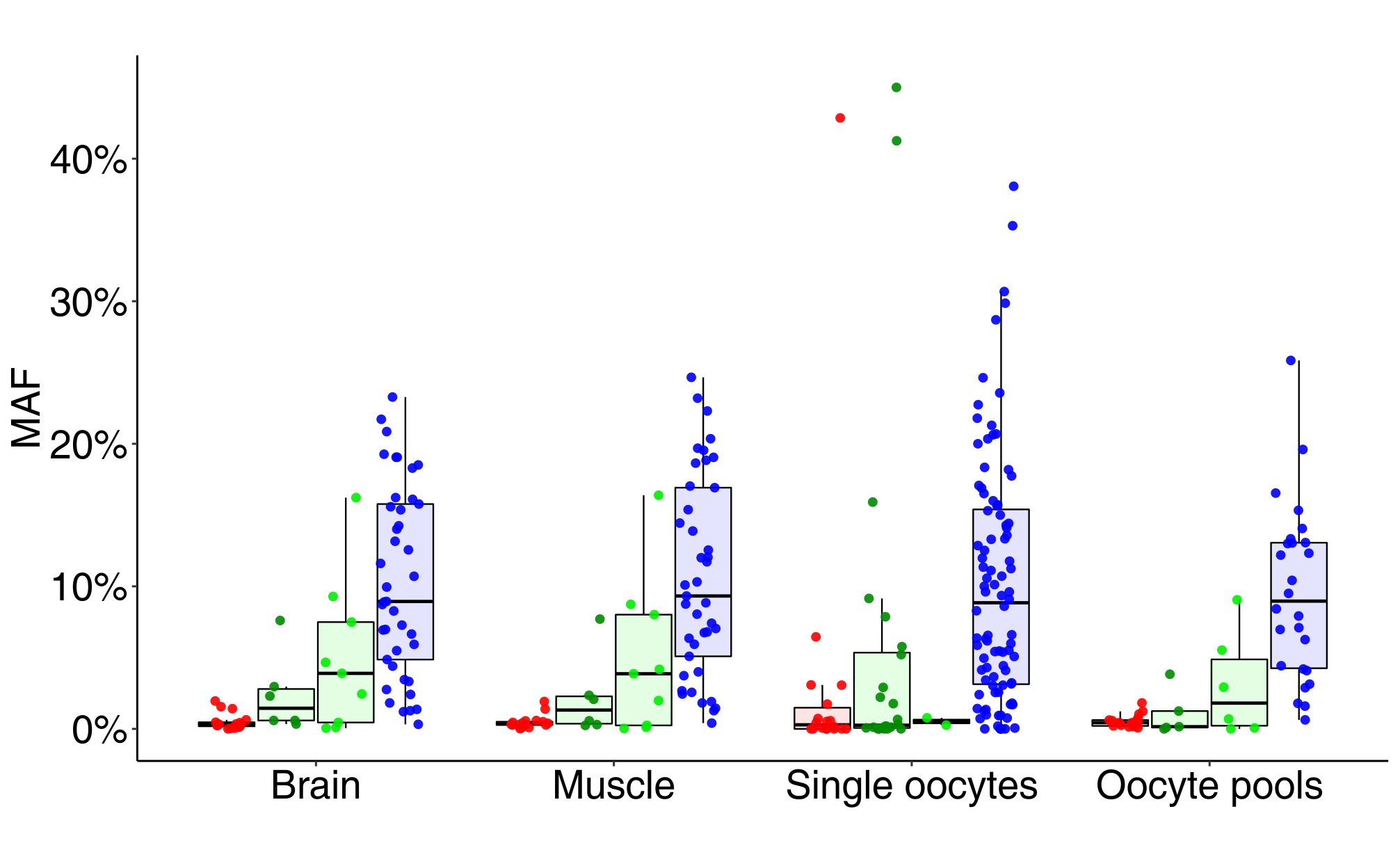

Supplement: S14 Fig — MAFs are shown for heteroplasmic sites separated by tissue (brain, muscle, single oocyte, and oocyte pool) and based on their occurrence in the pedigrees. Blue: heteroplasmies shared by several mothers and their pups of a pedigree (thus were likely also present in the grandmother); green: heteroplasmies shared by a mother and some of her pups (thus likely originated in the mother or grandmother), with dark green showing heteroplasmies observed in mothers (in which they are observed first) and light green showing heteroplasmies observed in pups (in which they are inherited); red: heteroplasmies present in both somatic tissues and oocytes of one or several pups but absent from their mothers (these mutations likely originated in the germline of the mothers and were inherited by the pups). The raw data for the information depicted in this figure are available at https://github.com/makovalab-psu/mouse-duplexSeq. MAF, minor allele frequency. (TIF) [file pbio.3000745.s014.tif]

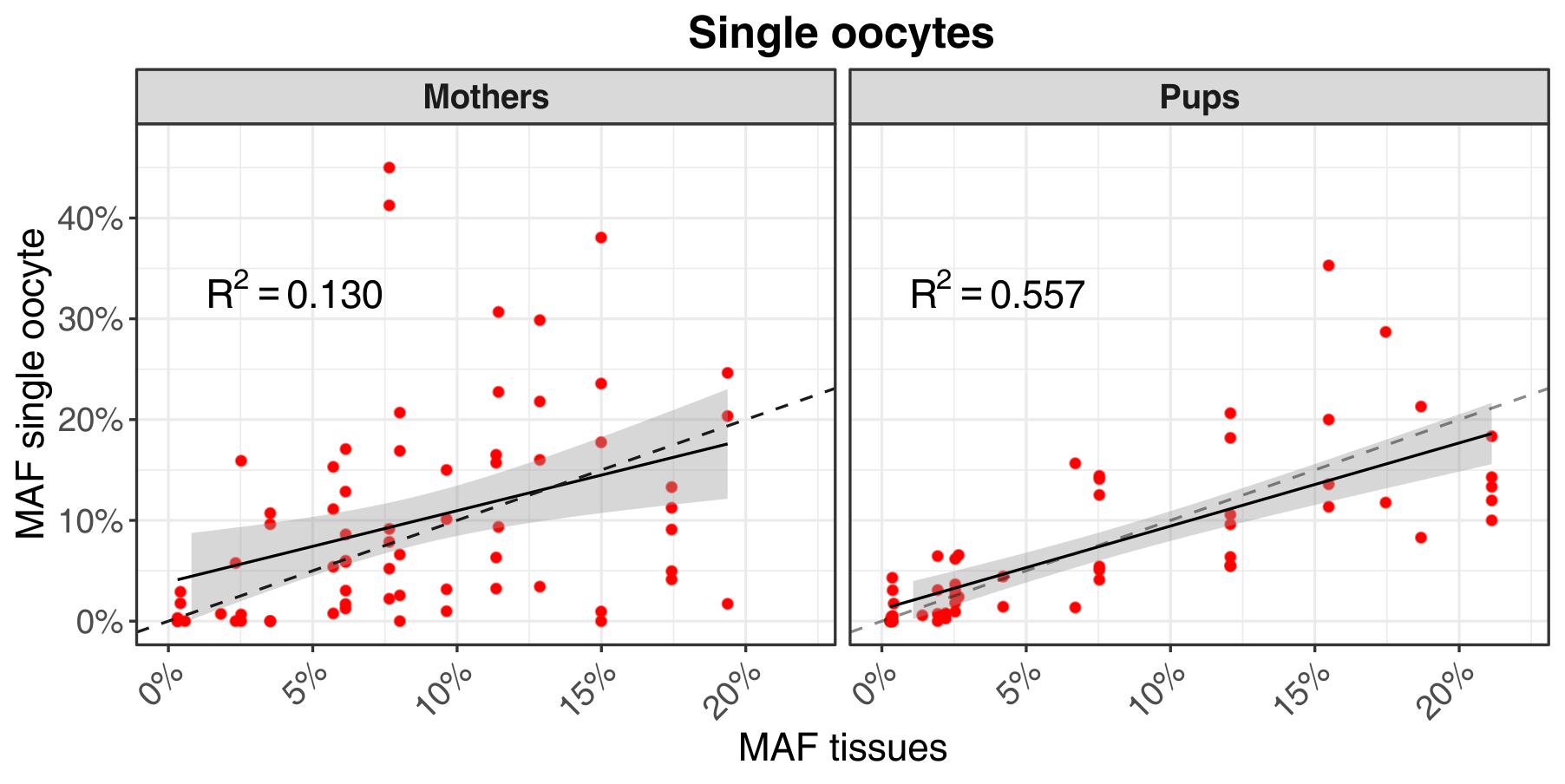

Supplement: S15 Fig — Heteroplasmy MAFs of single oocytes plotted against the average MAF in brain and muscle of the corresponding mouse. The raw data for the information depicted in this figure are available at https://github.com/makovalab-psu/mouse-duplexSeq. MAF, minor allele frequency. (TIF) [file pbio.3000745.s015.tif]

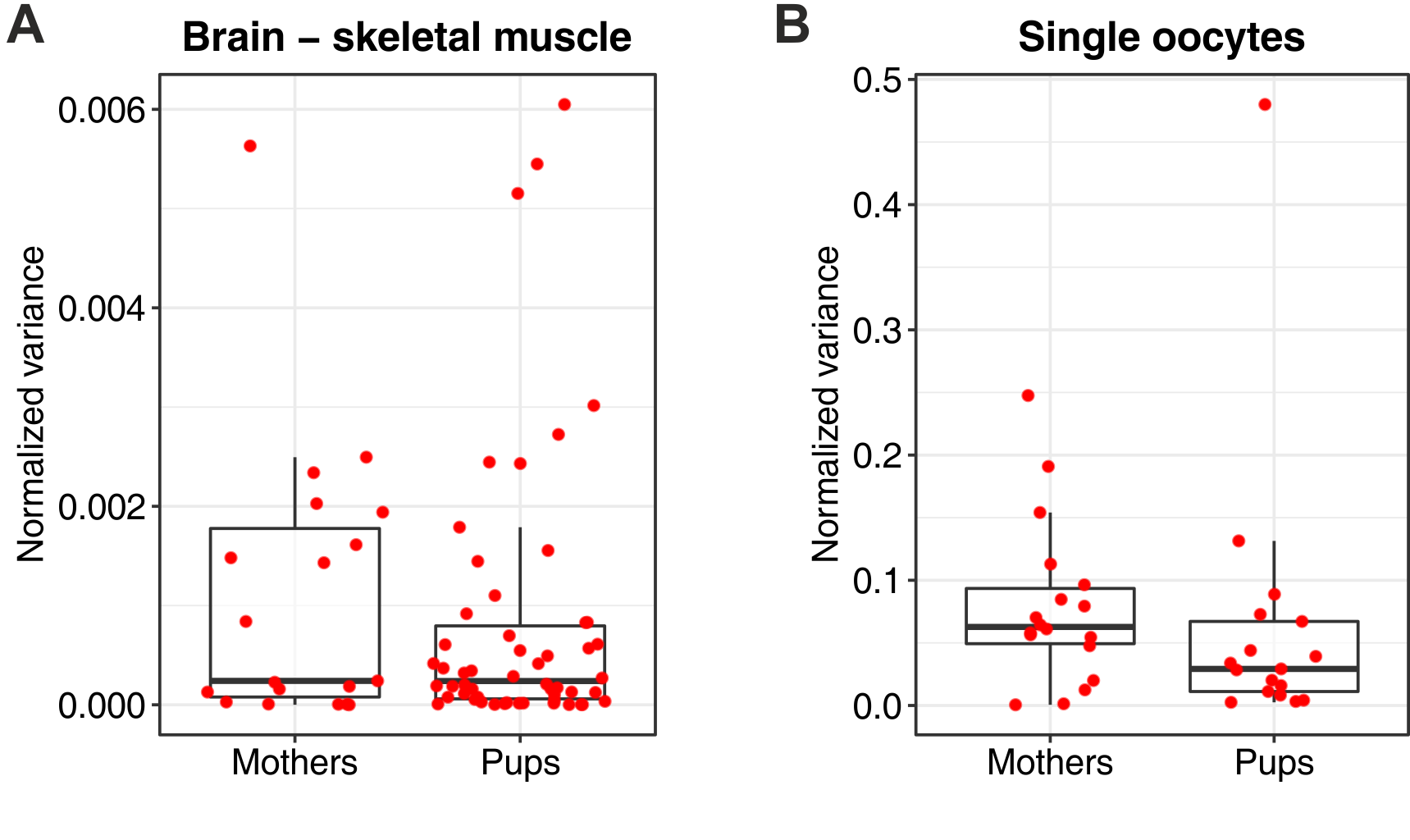

Supplement: S16 Fig — (A) Normalized variance (variance divided by p(1 − p), where p is the average allele frequency between somatic tissues or among single oocytes) of heteroplasmy MAFs in brain and muscle in pups and mothers. (B) Normalized variance of heteroplasmy MAFs in single oocytes of a mouse in pups and mothers. One-sided permutation test (medians; 10,000 permutations): p = 0.478 in (A) and p = 0.047 in (B). The raw data for the information depicted in this figure are available at https://github.com/makovalab-psu/mouse-duplexSeq. MAF, minor allele frequency. (TIF) [file pbio.3000745.s016.tif]

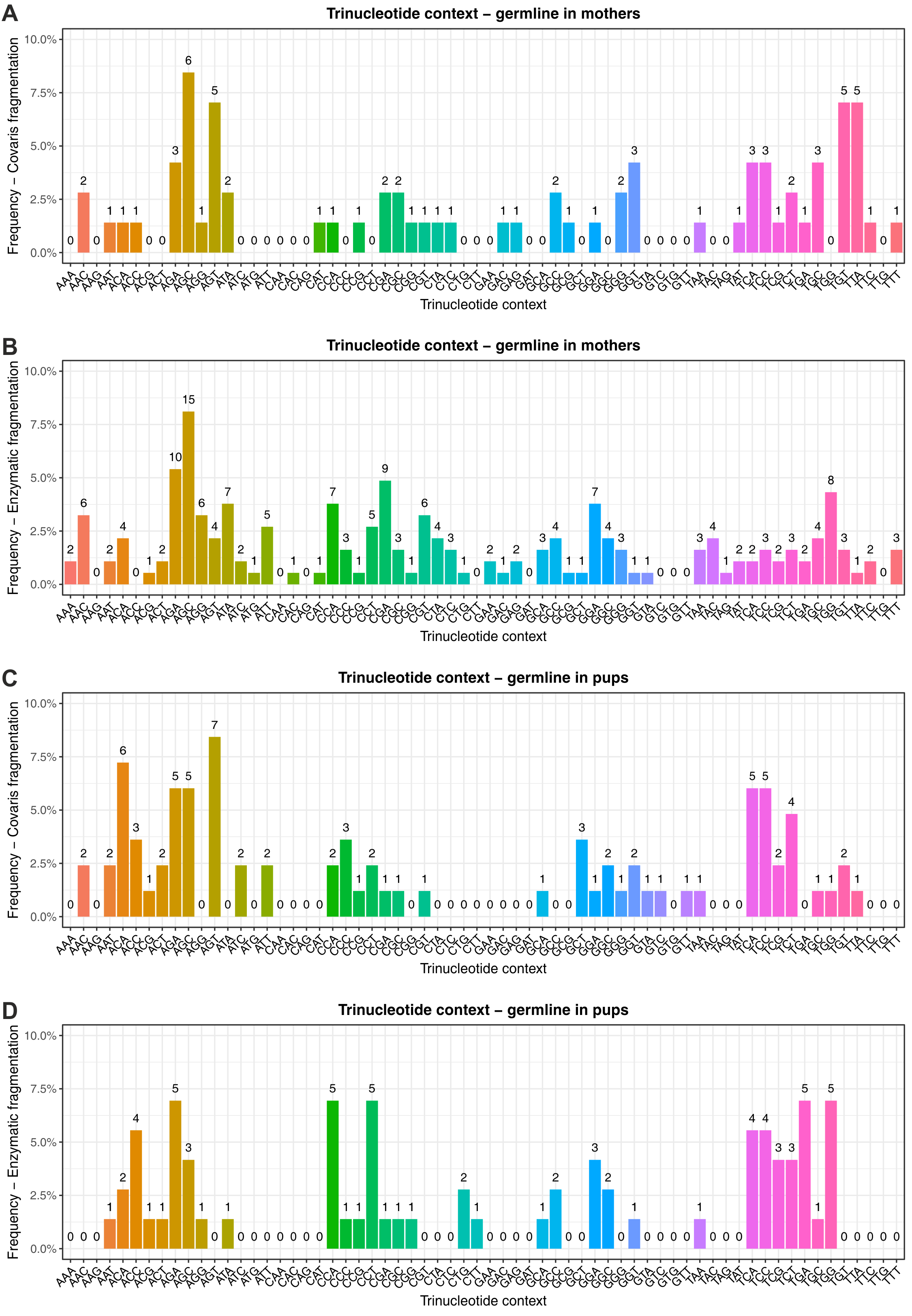

Supplement: S17 Fig — Because of the small number of mutations detected for each trinucleotide context in a sample type and age group, we do not have much power to detect differences between the used fragmentation methods. To statistically test for differences, we performed a Pearson’s chi-squared test of independence with Monte Carlo simulations (we can only compute p-values using simulations because the assumptions of the chi-squared approximation are not met). We did not observe any significant differences in the trinucleotide context of mutations between Covaris- and enzymatically sheared samples (p = 0.409 and p = 0.194 for mothers and pups, respectively). The raw data for the information depicted in this figure are available at https://github.com/makovalab-psu/mouse-duplexSeq. (TIF) [file pbio.3000745.s017.tif]

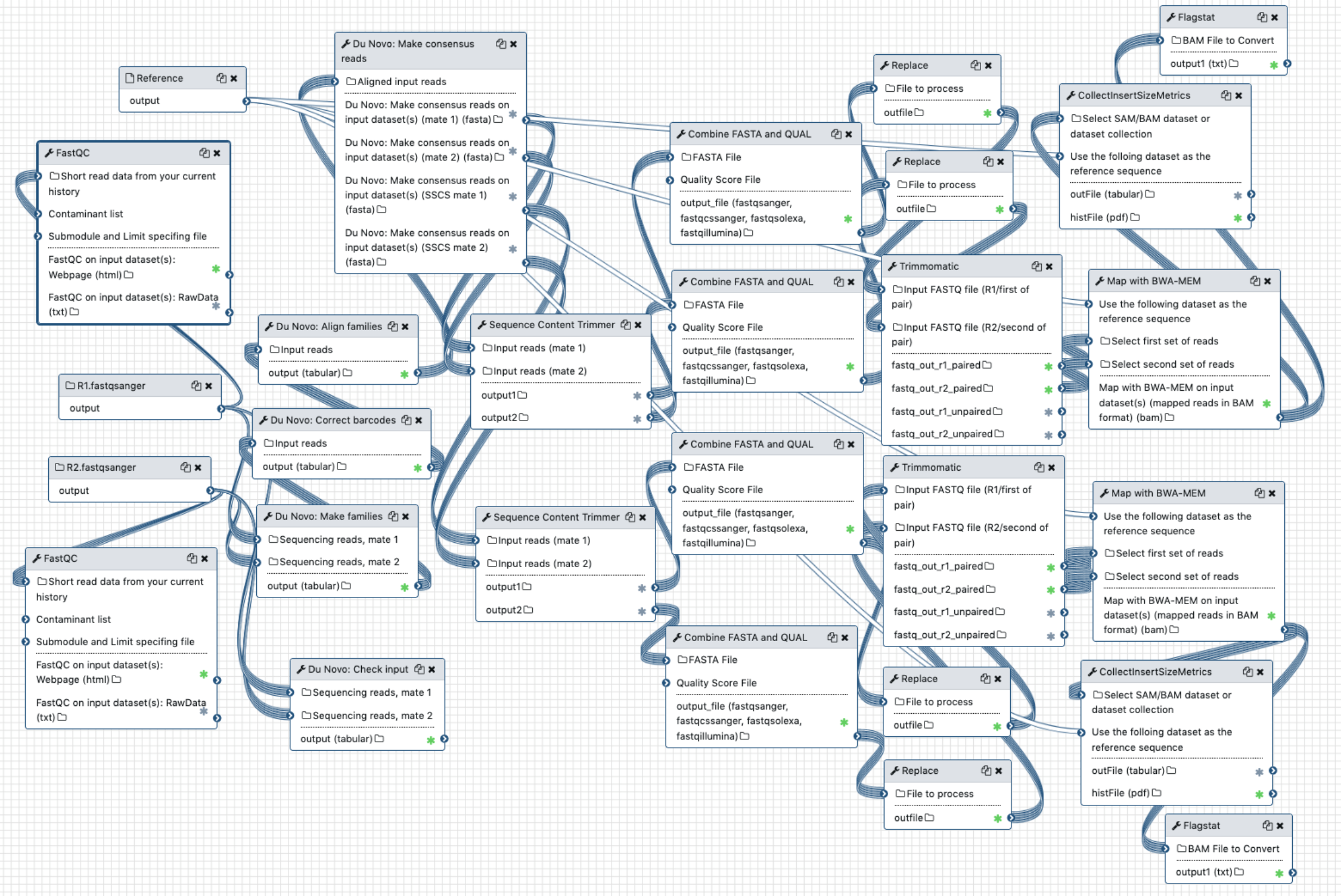

Supplement: S18 Fig — (TIF) [file pbio.3000745.s018.tif]

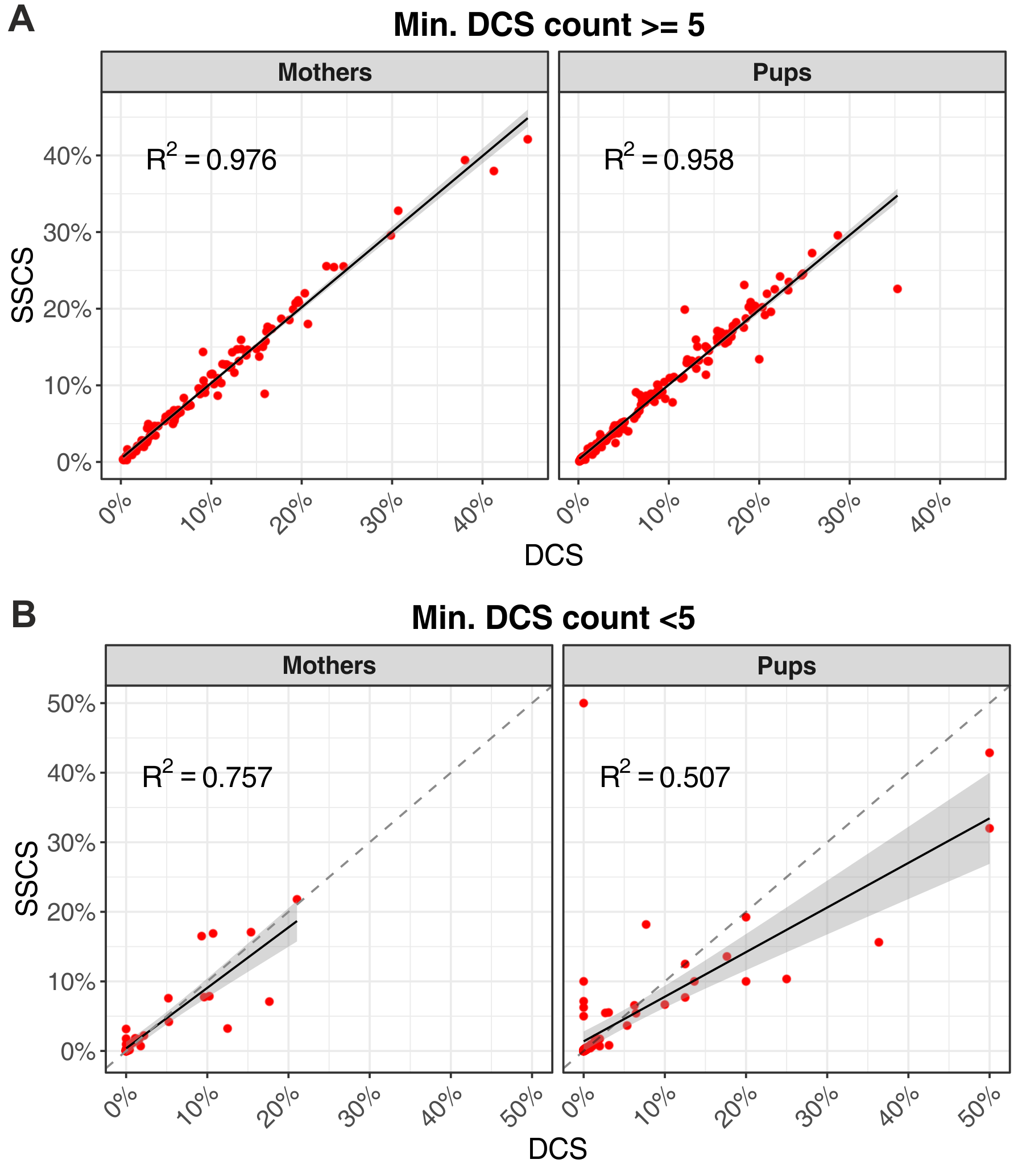

Supplement: S19 Fig — The x- and y-axes show MAFs for inherited heteroplasmies measured from DCSs and SSCSs, respectively. (A) When only considering heteroplasmies for which the minor allele was measured in at least five DCSs, measured frequencies correlate very well in mothers as well as pups. (B) Heteroplasmies for which the minor allele was measured in less than five DCSs show a lower correlation between DCS and SSCS, likely resulting from the large confidence interval of the DCS MAFs due to the small number of molecules analyzed. Because the number of measured molecules in duplex sequencing is at least 3× higher for SSCSs, heteroplasmy MAFs from SSCSs is likely more accurate. Therefore, for these sites, MAFs measured from SSCSs were used in subsequent analyses (as indicated in S3 Table). The raw data for the information depicted in this figure are available at https://github.com/makovalab-psu/mouse-duplexSeq. DCS, duplex consensus sequence; MAF, minor allele frequency; SSCS, single-strand consensus sequence. (TIF) [file pbio.3000745.s019.tif]
