# Supplementary material for: Age-related accumulation of de novo mitochondrial mutations in mammalian oocytes and somatic tissues
Source: PLoS Biol. 2020 Jul 15;18(7):e3000745. doi: 10.1371/journal.pbio.3000745 (PMC7363077; doi:10.1371/journal.pbio.3000745)
Supplement: S1 Note — (PDF) [file pbio.3000745.s040.pdf]

Note S1. Filtering of tissue-specific mutations based on their occurrence in the two pedigrees

Mutations (after excluding inherited heteroplasmic variants – found at 28 sites) can represent (1) tissue-specific *de novo* mutations, (2) early somatic mutations (present in both somatic tissues), or (3) variants segregating at low frequency in the two pedigrees. Since our focus was on the analysis of tissue-specific *de novo* mutations (i.e. '1'), we tried to exclude those other types of variants.

In the first step, we excluded mutations present in both analyzed somatic tissues (brain and muscle) of a mouse (n=128 – present at 64 positions) because they likely represent early somatic mutations (i.e. '2'). After filtering these variants, we retained 2,163 mutations (352, 422, 227, and 41 in brain, muscle, single oocytes, and oocyte pools of mothers, respectively, and 501, 454, 84, and 82 mutations in the same tissues of pups).

To distinguish between mutations of type '1' and '3', we next compared the occurrence of mutations present in more than one sample to what would be expected by random chance. To do so, we first estimated a rather conservative mutation frequency per nucleotide for our mouse samples (all tissues combined) by only including variants found in exactly one sample in a pedigree (n=1,759). Dividing this number by the total number of nucleotides sequenced in all our samples (3,411,410,700) we obtained a mutation frequency p of 5.16x10^{-7}.

We modeled the mutation process in a nucleotide of a single molecule as Bernoulli trial $X \sim B(1, p)$, where p is the mutation frequency per nucleotide estimated above. Assuming that mutations occur independently in different sequenced molecules, the number of mutations per nucleotide in a sample is $Y \sim B(d, p)$, where d is the average sequencing depth across all samples (1,284x). Hence the probability of calling a mutation in a sample (i.e. to identify it in at least one molecule) is $q = P\,(Y \geq 1) = 0.0006623247$. Given that the total number of samples included in the study is 188, and assuming independence, we obtain that the number of mutations per nucleotide called in this study is $W \sim B(188, q)$. Using this model we calculated that the probability of observing the same mutation in two or more samples by random chance is $P\,(W \geq 2) = 0.007105897$, and the expected number of mutations present at the same site in two or more samples is $0.007105897 \times 16{,}300 = \sim 116$, where 16,300 is the mtDNA length.

A comparison of the observed number of mutations occurring in two or more samples versus their expected number (calculated according to the model above, using our conservatively estimated mutation frequency which does not consider higher mutation frequencies in mother or in the D-loop) is shown in the Figure below.

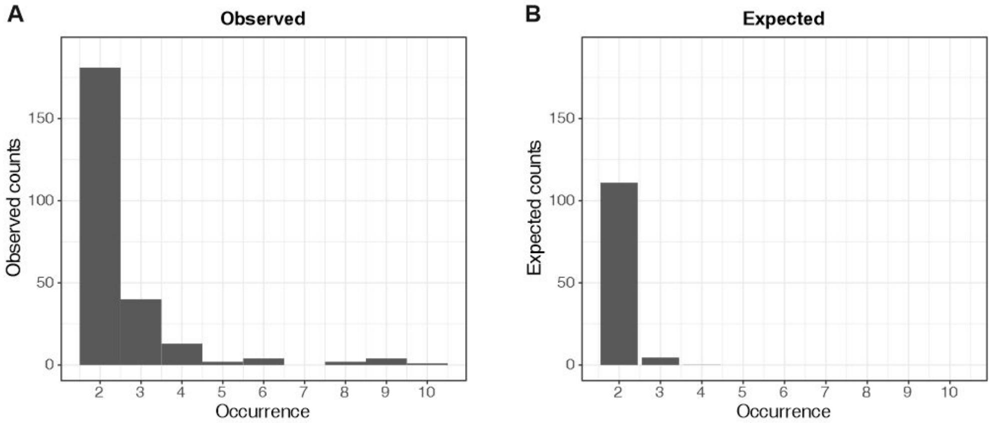

(A) observed versus (B) expected numbers of mutations observed in 2 or more samples in the two mouse pedigrees.

We next analyzed the distribution of sites for which mutations were found in two or more samples based on their occurrence within each of the two pedigrees (see Figure below).

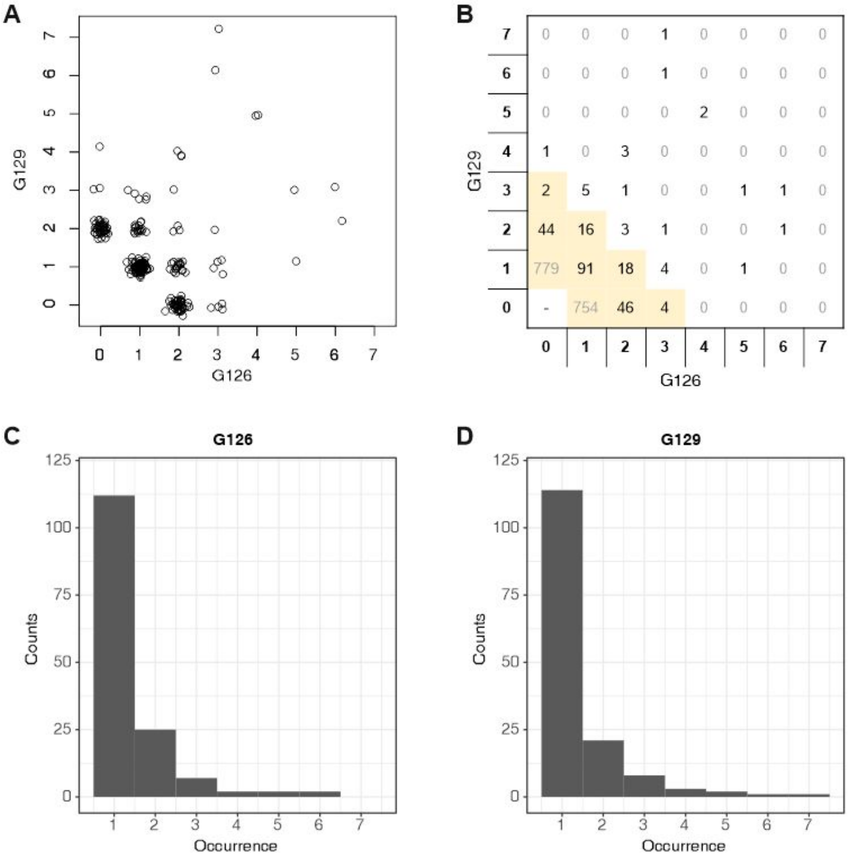

(A) Distribution of sites for which mutations were found in two or more samples based on their occurrence within each of the two pedigrees (G129 and G126). (B) Number of sites for which mutations were found in based on their occurrence within the two pedigrees. Mutations included in further analyses are highlighted in yellow. (C), (D) Mutations found in >1 samples, separately for the two pedigrees.

Based on these results, we decided to include all mutations in our analysis which were found in up to 3 samples in the two mouse pedigrees (2015 out of 2163 mutations). Those consisted of 1,533 mutations occurring in one sample, 362 mutations in two samples (occurring at 181 different sites),

and 120 mutations in three samples (occurring at 40 different sites). Mutations occurring more than three times at a site (n=148) are likely to represent either mutation hotspots or variants segregating at very low frequencies in a pedigree. However, since we cannot differentiate between these two possibilities, conservatively, we excluded these mutations from our analysis.

We next assessed whether the exclusion of such mutations had a qualitative effect on our major conclusions. Neither considering only mutations occurring in a single sample (1533 mutations) nor considering all observed mutations (2163 mutations) changed the observed trend of a higher mutation frequency in mothers compared to pups (see Figure below).

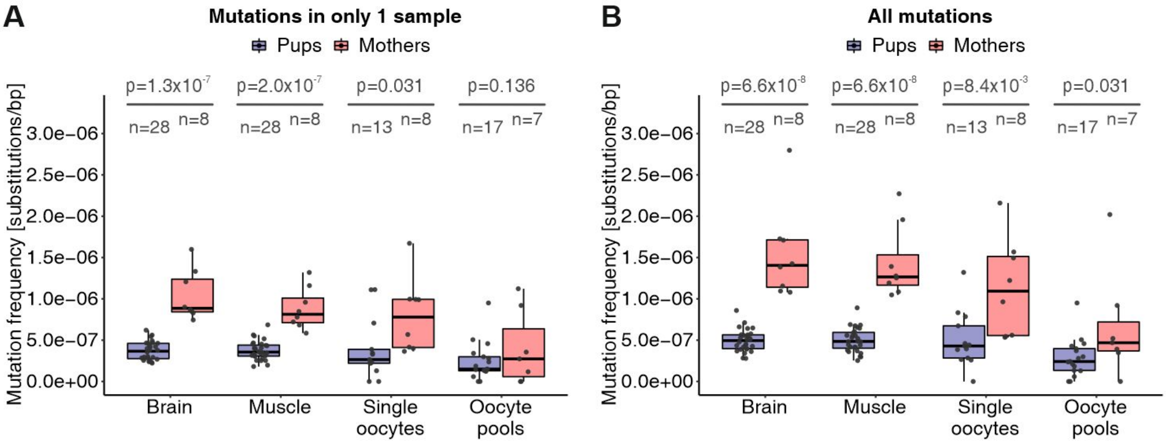

Accumulation of nucleotide substitutions with age. (A) Mutation frequencies observed in brain, muscle, single oocytes, and oocyte pools of mothers compared to pups based on mutations found in a single sample (1533 mutations). **(B)** Mutation frequencies observed in brain, muscle, single oocytes, and oocyte pools of mothers compared to pups based on all observed mutations (2163 mutations). Permutation test p-values are indicated (one-sided test based on medians, testing if mutations are higher in mothers than pups; 100,000,000 permutations; corrected for multiple testing); n: sample size. In the calculation of mutation frequencies for single oocytes, the numbers of mutations and sequenced nucleotides were combined across all oocytes measured for a mouse.

The majority of excluded mutations were located in the D-loop, which was shown to have a higher mutation frequency compared to all other mtDNA regions (see Figure below).

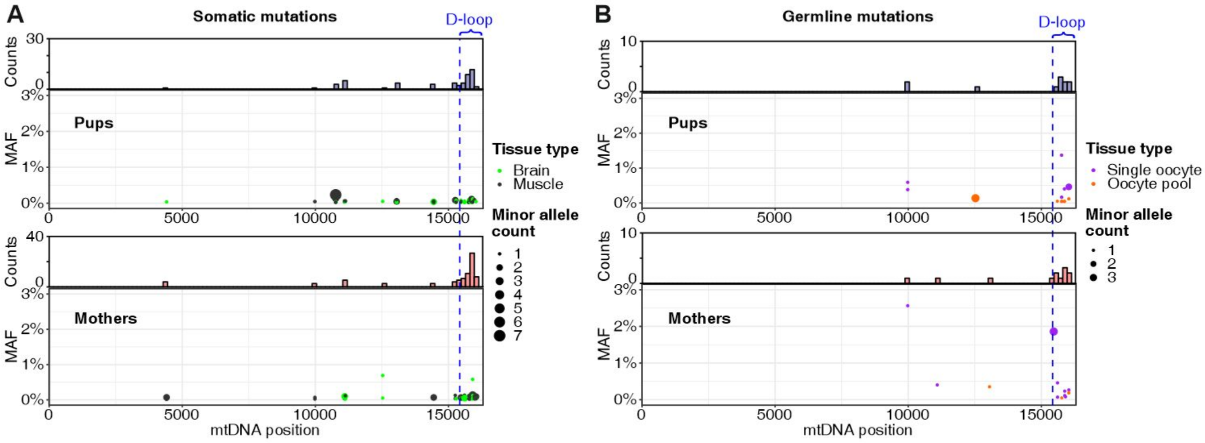

Mutations excluded from the analysis shown in the main text, which were present in more than three samples. (A) Distribution of somatic mutations in the mitochondrial genome. **(B)** Distribution of germline mutations in the mitochondrial genome. Dot sizes represent the number of molecules (DCS) in which the mutation was observed.
