# Supplementary material for: Age-related accumulation of de novo mitochondrial mutations in mammalian oocytes and somatic tissues
Source: PLoS Biol. 2020 Jul 15;18(7):e3000745. doi: 10.1371/journal.pbio.3000745 (PMC7363077; doi:10.1371/journal.pbio.3000745)
Supplement: S2 Note — (PDF) [file pbio.3000745.s041.pdf]

**Note S2. Selection analysis - hN/hS statistics**

Code to test for selection (mutations in brain of mothers are used as an example)

# Selection test - mouse (brain - mothers)

Arslan Zaidi, modified by Barbara Arbeithuber

8/11/2019

Introduction

The goal here is to test whether the distribution of mutations observed in our duplex sequencing data exhibits signatures of purifying selection. To test for this, we will use the hN/hS statistic (Li et al. 2015), which is similar in concept to the $d_N/d_S$ statistic.

Analysis

First, we load all the required packages.

```
## Loading required package: BiocGenerics

## Loading required package: parallel

##
## Attaching package: 'BiocGenerics'

## The following objects are masked from 'package:parallel':
##
##     clusterApply, clusterApplyLB, clusterCall, clusterEvalQ,
##     clusterExport, clusterMap, parApply, parCapply, parLapply,
##     parLapplyLB, parRapply, parSapply, parSapplyLB

## The following objects are masked from 'package:stats':
##
##     IQR, mad, sd, var, xtabs

## The following objects are masked from 'package:base':
##
##     anyDuplicated, append, as.data.frame, basename, cbind,
##     colnames, dirname, do.call, duplicated, eval, evalq, Filter,
##     Find, get, grep, grepl, intersect, is.unsorted, lapply, Map,
##     mapply, match, mget, order, paste, pmax, pmax.int, pmin,
##     pmin.int, Position, rank, rbind, Reduce, rownames, sapply,
##     setdiff, sort, table, tapply, union, unique, unsplit, which,
##     which.max, which.min

## Loading required package: S4Vectors

## Loading required package: stats4

##
## Attaching package: 'S4Vectors'

## The following objects are masked from 'package:data.table':
##
##     first, second
```

```
## The following object is masked from 'package:base':
##
##     expand.grid

## Loading required package: IRanges

##
## Attaching package: 'IRanges'

## The following object is masked from 'package:data.table':
##
##     shift

## Loading required package: XVector

##
## Attaching package: 'Biostrings'

## The following object is masked from 'package:ape':
##
##     complement

## The following object is masked from 'package:base':
##
##     strsplit

##
## Attaching package: 'dplyr'

## The following objects are masked from 'package:Biostrings':
##
##     collapse, intersect, setdiff, setequal, union

## The following object is masked from 'package:XVector':
##
##     slice

## The following objects are masked from 'package:IRanges':
##
##     collapse, desc, intersect, setdiff, slice, union

## The following objects are masked from 'package:S4Vectors':
##
##     first, intersect, rename, setdiff, setequal, union

## The following objects are masked from 'package:BiocGenerics':
##
##     combine, intersect, setdiff, union

## The following objects are masked from 'package:data.table':
##
##     between, first, last

## The following objects are masked from 'package:stats':
##
##     filter, lag

## The following objects are masked from 'package:base':
##
##     intersect, setdiff, setequal, union

##
## Attaching package: 'tidyr'
```

```
## The following object is masked from 'package:S4Vectors':
##
##     expand

## here() starts at /Users/babsi/duplex_analysis/2019-08-09_selection_Arslan
```

Load major and minor alleles observed in Duplex sequencing. Remove all sites where heteroplasmies were not observed i.e. minor allele is ".". Assume major allele is the ancestral state and the minor allele is the mutant allele.

```
dat<-fread(("Br_mom_full.txt "),header=T)
hq<-dat%>%
  filter(minor!=".")

head(hq)

##   position major minor
## 1       75     T     G
## 2       85     G     A
## 3      178     G     A
## 4      353     G     T
## 5      407     G     T
## 6      420     G     A
```

Recode major/minor/sequence context to pyrimidines.

```
#recode alleles so ancestral allele is a pyrimidine - for mutational signatures
hq$major.py<-NA
hq$minor.py<-NA
#pb<-txtProgressBar(min=1,max=nrow(hq),style=3)
for(i in 1:nrow(hq)){
  if(hq$major[i]%in%c("G","A")){
    hq$major.py[i]<-as.character(Biostrings::complement(DNAString(hq$major[i])))
    hq$minor.py[i]<-as.character(Biostrings::complement(DNAString(hq$minor[i])))
  }
  if(hq$major[i]%in%c("C","T")){
    hq$major.py[i]<-hq$major[i]
    hq$minor.py[i]<-hq$minor[i]
  }
  #setTxtProgressBar(pb,i)
}
```

Load full mtDNA mouse reference genome and genbank file.

```
#read reference genome reference
ref<-read.dna(("mtDNA_Mouse.fasta"),format="fasta")

#mt.code
mt.code<-getGeneticCode("2")

#load genbank file - ref annotation for genes
genedb<-fread(("mtDNA_mouse.gb.TXT"),header=T)
```

Annotate each mutation by which gene it is present in

```
hq$gene<-NA
for(i in 1:nrow(genedb)){
  hq$gene[which(hq$position>=genedb$Start[i] &
hq$position<=genedb$Stop[i])]<-genedb$Gene[i]
}
```

Annotate mutations by whether they are synonymous/non-synonymous and by their fold degeneracy etc. This will be useful later for the hN/hS test.

```
#write function to return fold degeneracy for a site in a given codon
fold.degeneracy<-function(codon,site){
  aa<-Biostrings::translate(DNAString(codon),mt.code)
  codon<-unlist(strsplit(codon,split=""))
  nucs<-setdiff(c("a","c","g","t"),codon[site])
  s<-1
  for(l in 1:3){
    mut.codon<-codon
    mut.codon[site]<-nucs[l]
    mt.aa<-translate(DNAString(paste(mut.codon,collapse="")),mt.code)
    if(mt.aa==aa){s<-s+1}else{s<-s}
  }
  #if all 3 substitutions are nonsynonymous, site is 4-fold denegerate
  return(s)
}

#calculate a number of things (e.g. fold degeneracy etc.)
hq$hq.gene.pos<-NA
hq$hq.codon.pos<-NA
hq$major.codon<-NA
hq$minor.codon<-NA
hq$major.aa<-NA
hq$minor.aa<-NA
hq$fold.d<-NA
pb<-txtProgressBar(min=1,max=nrow(hq),style=3)
for(i in 1:nrow(hq)){
  gene.name<-hq$gene[i]
  if(gene.name%in%genedb$Gene){
    gene.start<-genedb$Start[which(genedb$Gene==hq$gene[i])]
    gene.end<-genedb$Stop[which(genedb$Gene==hq$gene[i])]
    gene.seq<-as.character(ref[(gene.start):(gene.end)])
    gene.strand<-genedb$Strand[which(genedb$Gene==hq$gene[i])]
    #hq.gene.pos<-hq$position[i]-(gene.start-1)
    if(gene.strand==1){
      hq.gene.pos<-hq$position[i]-(gene.start-1)
      if(hq.gene.pos%%3==0){
        hq.codon<-hq.gene.pos/3
        hq.codon.pos<-3
        hq$hq.codon.pos[i]<-3
        hq.codon.seq<-gene.seq[c(hq.gene.pos-2,hq.gene.pos-1,hq.gene.pos)]
        fold.d<-fold.degeneracy(paste(hq.codon.seq,collapse=""),hq.codon.pos)
      }
      if(hq.gene.pos%%3==1){
        hq.codon<-ceiling(hq.gene.pos/3)
        hq.codon.pos<-1
        hq$hq.codon.pos[i]<-1
        hq.codon.seq<-gene.seq[c(hq.gene.pos,hq.gene.pos+1,hq.gene.pos+2)]
        fold.d<-fold.degeneracy(paste(hq.codon.seq,collapse=""),hq.codon.pos)
      }
      if(hq.gene.pos%%3==2){
        hq.codon<-ceiling(hq.gene.pos/3)
        hq.codon.pos<-2
        hq$hq.codon.pos[i]<-2
        hq.codon.seq<-gene.seq[c(hq.gene.pos-1,hq.gene.pos,hq.gene.pos+1)]
        fold.d<-fold.degeneracy(paste(hq.codon.seq,collapse=""),hq.codon.pos)
      }
      hq.major.seq<-hq.minor.seq<-hq.codon.seq
      hq.major.seq[hq.codon.pos]<-as.character(hq$major[i])
      hq.minor.seq[hq.codon.pos]<-as.character(hq$minor[i])

hq.major.aa<-as.character(translate(DNAString(paste(hq.major.seq,collapse="")),mt.code))

hq.minor.aa<-as.character(translate(DNAString(paste(hq.minor.seq,collapse="")),mt.code))

    }
    if(gene.strand== -1){
```

```
      gene.seq<-reverseComplement(DNAString(paste(gene.seq,collapse="")))
      gene.seq<-unlist(strsplit(as.character(gene.seq),split=""))
      hq.gene.pos<-gene.end-(hq$position[i]-1)
      if(hq.gene.pos%%3==0){
        hq.codon<-hq.gene.pos/3
        hq.codon.pos<-3
        hq$hq.codon.pos[i]<-3
        hq.codon.seq<-gene.seq[c(hq.gene.pos-2,hq.gene.pos-1,hq.gene.pos)]
        fold.d<-fold.degeneracy(paste(hq.codon.seq,collapse=""),hq.codon.pos)
      }
      if(hq.gene.pos%%3==1){
        hq.codon<-ceiling(hq.gene.pos/3)
        hq.codon.pos<-1
        hq$hq.codon.pos[i]<-1
        hq.codon.seq<-gene.seq[c(hq.gene.pos,hq.gene.pos+1,hq.gene.pos+2)]
        fold.d<-fold.degeneracy(paste(hq.codon.seq,collapse=""),hq.codon.pos)
      }
      if(hq.gene.pos%%3==2){
        hq.codon<-ceiling(hq.gene.pos/3)
        hq.codon.pos<-2
        hq$hq.codon.pos[i]<-2
        hq.codon.seq<-gene.seq[c(hq.gene.pos-1,hq.gene.pos,hq.gene.pos+1)]
        fold.d<-fold.degeneracy(paste(hq.codon.seq,collapse=""),hq.codon.pos)
      }
      hq.major.seq<-hq.minor.seq<-hq.codon.seq
      major<-Biostrings::complement(DNAString(hq$major[i]))
      minor<-Biostrings::complement(DNAString(hq$minor[i]))
      hq.major.seq[hq.codon.pos]<-as.character(major)
      hq.minor.seq[hq.codon.pos]<-as.character(minor)

hq.major.aa<-as.character(translate(DNAString(paste(hq.major.seq,collapse="")),mt.code))

hq.minor.aa<-as.character(translate(DNAString(paste(hq.minor.seq,collapse="")),mt.code))
    }
    hq$fold.d[i]<-fold.d
    hq$hq.gene.pos[i]<-hq.gene.pos
    hq$major.codon[i]<-paste(tolower(hq.major.seq),collapse="")
    hq$minor.codon[i]<-paste(tolower(hq.minor.seq),collapse="")
    hq$major.aa[i]<-hq.major.aa
    hq$minor.aa[i]<-hq.minor.aa
  }
  #setTxtProgressBar(pb,i)
}

hq$syn<-NA
hq$syn[which(hq$major.aa==hq$minor.aa)]<-"syn"
hq$syn[which(hq$major.aa!=hq$minor.aa)]<-"nsyn"
```

Summarize the total number of mutations observed in each gene as well as overall across all genes

```
#calculate total number of syn/nsyn mutations in every gene
dhq.syn<-as.data.frame(hq%>%
                         filter(gene%in%genedb$Gene)%>%
                         group_by(gene,syn)%>%
                         summarize(nhets=length(position)))

#make sure each gene is represented at least twice (once for synonymous and once for
non-synonymous numbers)
table(dhq.syn$gene)

##
## ATP6 ATP8 COX1 COX2 COX3 CYTB  ND1  ND2  ND3  ND4 ND4L  ND5  ND6
##    2    2    2    2    2    2    2    2    2    2    2    2    1
```

```r
#add dummy rows with 0 values for genes without mutations; Here: ND6 (syn)
dhq.syn<-rbind(dhq.syn,data.frame(gene=c("ND6"),
                                  syn=c("syn"),
                                  nhets=c(0)))
#add numbers for entire genome for plotting
ref_syn=sum(dhq.syn$nhets[which(dhq.syn$syn=="syn")])
ref_nsyn=sum(dhq.syn$nhets[which(dhq.syn$syn=="nsyn")])

dhq.syn<-rbind(dhq.syn,
               data.frame(gene=c("ref","ref"),
                          syn=c("nsyn","syn"),
                          nhets=c(ref_nsyn,ref_syn)))

#calculate total number of mutations by mutation type in coding sequences
hq.spectrum<-hq%>%
  filter(gene%in%genedb$Gene)%>%
  group_by(gene,major,minor)%>%
  summarize(nhets=length(position))
```

Now calculate no. of syn/nsyn *sites* in each gene and in the whole coding sequence.

```r
#Create empty columns to store the number of syn/nsyn sites per gene
genedb$syn.sites<-NA
genedb$nsyn.sites<-NA

#write function to calculate nsyn sites and syn sites for a single codon
nei.sites<-function(codon){
  aa<-Biostrings::translate(DNAString(codon),mt.code)
  codon<-unlist(strsplit(codon,split=""))
  s<-0
  for(k in 1:3){
    nucs<-setdiff(c("a","c","g","t"),codon[k])
    for(l in 1:3){
      mut.codon<-codon
      mut.codon[k]<-nucs[l]
      mt.aa<-Biostrings::translate(DNAString(paste(mut.codon,collapse="")),mt.code)
      if(mt.aa==aa){s<-s+1/3}else{s<-s}
    }
  }
  n<-3-s
  return(c(syn.sites=s,nsyn.sites=n))
}

#apply this function to all codons for every gene
for(i in 1:nrow(genedb)){
  gene.start<-genedb$Start[i]
  gene.end<-genedb$Stop[i]
  gene.seq<-as.character(ref[c(gene.start:gene.end)])
  if(genedb$Strand[i]==1){gene.seq<-gene.seq}
  if(genedb$Strand[i]==-1){
    gene.seq<-tolower(
      unlist(
        strsplit(
          as.character(
            reverseComplement(
              DNAString(
                paste(gene.seq,collapse=""))))),split="")))}
  if(length(gene.seq)%%3==0){
    gene.seq<-gene.seq
  }
  if(length(gene.seq)%%3==1){
    gene.seq<-c(gene.seq,"a","a")
  }
  if(length(gene.seq)%%3==2){
    gene.seq<-c(gene.seq,"a")
```

```
  }
  gene.codons<-seqinr::splitseq(gene.seq,0,3)
  #print(genedb2$gene[i])

  nsites<-sapply(gene.codons,nei.sites)
  nsites<-apply(nsites,1,sum)
  genedb$syn.sites[i]<-nsites[1]
  genedb$nsyn.sites[i]<-nsites[2]
  genedb$seq[i]<-paste(gene.seq,collapse="")
}

#convert genedb to long format for plotting and merging with hq object
dgenedb.syn<-genedb%>%
  select(Gene,syn.sites,nsyn.sites)%>%
  melt(id.vars="Gene",variable.name="synonymity",value.name="nsites")%>%
  mutate(syn=case_when(synonymity=="syn.sites"~"syn",
                       synonymity=="nsyn.sites"~"nsyn"))

#add ref to dgene.db
ref_ssites<-sum(dgenedb.syn$nsites[which(dgenedb.syn$syn=="syn")])
ref_nsites<-sum(dgenedb.syn$nsites[which(dgenedb.syn$syn=="nsyn")])

dgenedb.syn<-rbind(dgenedb.syn,data.frame(Gene=c("ref","ref"),
                                          synonymity=c("nsyn.sites","syn.sites"),
                                          nsites=c(ref_nsites,ref_ssites),
                                          syn=c("nsyn","syn")))
colnames(dhq.syn)[1]<-"Gene"
#merged dhq.syn and dgenedb.syn
dmerged<-merge(dhq.syn,dgenedb.syn,by=c("Gene","syn"))


ref_ssites

## [1] 3020.333

ref_nsites

## [1] 8388.667
```

Calculate *observed* values of hN/hS for each gene. This is done by dividing the number of observed syn (nsyn) mutations by the number of syn (nsyn) sites.

```
#calculate observed hN/hS
dmerged<-dmerged%>%
  mutate(prop=nhets/nsites)%>%
  mutate(k=prop)

mdmerged<-dmerged%>%
  dcast(Gene~syn,value.var="k")%>%
  mutate(hn_hs=nsyn/syn)

head(dmerged, 30)

##    Gene  syn nhets synonymity     nsites        prop           k
## 1  ATP6 nsyn     5 nsyn.sites  491.00000 0.010183299 0.010183299
## 2  ATP6  syn     3  syn.sites  190.00000 0.015789474 0.015789474
## 3  ATP8 nsyn     3 nsyn.sites  151.33333 0.019823789 0.019823789
## 4  ATP8  syn     1  syn.sites   52.66667 0.018987342 0.018987342
## 5  COX1 nsyn    19 nsyn.sites 1144.66667 0.016598719 0.016598719
## 6  COX1  syn     7  syn.sites  400.33333 0.017485429 0.017485429
## 7  COX2 nsyn     6 nsyn.sites  509.00000 0.011787819 0.011787819
## 8  COX2  syn     2  syn.sites  175.00000 0.011428571 0.011428571
## 9  COX3 nsyn    20 nsyn.sites  589.00000 0.033955857 0.033955857
## 10 COX3  syn     3  syn.sites  197.00000 0.015228426 0.015228426
## 11 CYTB nsyn    23 nsyn.sites  848.00000 0.027122642 0.027122642
```

```
## 12 CYTB  syn     7  syn.sites  298.00000 0.023489933 0.023489933
## 13  ND1 nsyn    15 nsyn.sites  699.66667 0.021438780 0.021438780
## 14  ND1  syn     6  syn.sites  257.33333 0.023316062 0.023316062
## 15  ND2 nsyn    13 nsyn.sites  746.00000 0.017426273 0.017426273
## 16  ND2  syn     4  syn.sites  292.00000 0.013698630 0.013698630
## 17  ND3 nsyn     3 nsyn.sites  256.33333 0.011703511 0.011703511
## 18  ND3  syn     1  syn.sites   91.66667 0.010909091 0.010909091
## 19  ND4 nsyn    21 nsyn.sites 1000.33333 0.020993002 0.020993002
## 20  ND4  syn     4  syn.sites  379.66667 0.010535558 0.010535558
## 21 ND4L nsyn     1 nsyn.sites  215.00000 0.004651163 0.004651163
## 22 ND4L  syn     2  syn.sites   82.00000 0.024390244 0.024390244
## 23  ND5 nsyn    26 nsyn.sites 1344.00000 0.019345238 0.019345238
## 24  ND5  syn     4  syn.sites  480.00000 0.008333333 0.008333333
## 25  ND6 nsyn     4 nsyn.sites  394.33333 0.010143702 0.010143702
## 26  ND6  syn     0  syn.sites  124.66667 0.000000000 0.000000000
## 27  ref nsyn   159 nsyn.sites 8388.66667 0.018954144 0.018954144
## 28  ref  syn    44  syn.sites 3020.33333 0.014567928 0.014567928
```

```
head(mdmerged, 30)
```

```
##    Gene        nsyn         syn     hn_hs
## 1  ATP6 0.010183299 0.015789474 0.6449423
## 2  ATP8 0.019823789 0.018987342 1.0440529
## 3  COX1 0.016598719 0.017485429 0.9492886
## 4  COX2 0.011787819 0.011428571 1.0314342
## 5  COX3 0.033955857 0.015228426 2.2297680
## 6  CYTB 0.027122642 0.023489933 1.1546496
## 7   ND1 0.021438780 0.023316062 0.9194855
## 8   ND2 0.017426273 0.013698630 1.2721180
## 9   ND3 0.011703511 0.010909091 1.0728218
## 10  ND4 0.020993002 0.010535558 1.9925858
## 11 ND4L 0.004651163 0.024390244 0.1906977
## 12  ND5 0.019345238 0.008333333 2.3214286
## 13  ND6 0.010143702 0.000000000       Inf
## 14  ref 0.018954144 0.014567928 1.3010871
```

Generate neutral distribution of hN/hS by bootstrapping from the observed mutation spectrum 100 times. For example, if there are 10 C>T mutations, mutate 10 Cs chosen uniformly at random to Ts. Then calculate hN/hS.

```
#define function to bootstrap for each gene separately
dnds.boot<-function(gn){
  if(gn=="ref"){
    total_mutations=sum(hq.spectrum$nhets)
    #concatenate all coding sequences
    gene_seq=unlist(strsplit(paste(genedb$seq,collapse=""),split=""))
    #create mutated sequence
    mut_seq=gene_seq
    hq_spec=hq.spectrum%>%
      group_by(major,minor)%>%
      summarize(nhets=sum(nhets))
    syn_sites=sum(genedb$syn.sites)
    nsyn_sites=sum(genedb$nsyn.sites)

  }
  else{
    db<-genedb%>%
      filter(Gene==gn)

    hq_spec=hq.spectrum%>%
      filter(gene==gn)

    total_mutations=sum(hq_spec$nhets)

    gene_seq<-unlist(strsplit(db$seq,split=""))
    mut_seq=gene_seq
```

```
    nsyn_sites=db$nsyn.sites
    syn_sites=db$syn.sites
  }

  for(i in 1:nrow(hq_spec)){
    sites=which(gene_seq==tolower(hq_spec$major[i]))
    nmutations=hq_spec$nhets[i]
    #randomly sample these many sites to mutate
    mut_sites=sample(sites,nmutations)
    mut_seq[mut_sites]=tolower(hq_spec$minor[i])
  }

  anc_codons<-seqinr::splitseq(gene_seq,0,3)
  mut_codons<-seqinr::splitseq(mut_seq,0,3)

  #dissimilar codons

  anc_mismatch=paste(anc_codons[which(anc_codons!=mut_codons)],collapse="")
  mut_mismatch=paste(mut_codons[which(anc_codons!=mut_codons)],collapse="")


  anc_aa<-unlist(strsplit(as.character(translate(DNAString(anc_mismatch),genetic.code =
mt.code)),split=""))
  mut_aa<-unlist(strsplit(as.character(translate(DNAString(mut_mismatch),genetic.code =
mt.code)),split=""))

  nsyn=length(which(anc_aa!=mut_aa))
  syn=total_mutations-nsyn

  hn=nsyn/nsyn_sites
  hs=syn/syn_sites
  hn_hs=hn/hs
  if(hs==0){return(NA)}else{return(hn_hs)}

}


#calculate neutral distribution for every gene and the whole ref coding sequence
#apply function to each gene separately.
gene_list=c("ref",unique(genedb$Gene))
dnds_bootmat=matrix(NA,nrow=100,ncol=length(gene_list))
for(j in 1:length(gene_list)){
  dnds_bootmat[,j]<-replicate(100,dnds.boot(gene_list[j]))
  print(gene_list[j])
}

## [1] "ref"
## [1] "ND1"
## [1] "ND2"
## [1] "COX1"
## [1] "COX2"
## [1] "ATP8"
## [1] "ATP6"
## [1] "COX3"
## [1] "ND3"
## [1] "ND4L"
## [1] "ND4"
## [1] "ND5"
## [1] "ND6"
## [1] "CYTB"
```

Plot the neutral distribution of $h_N/h_S$ (black) for each gene separately and the whole reference genome against the observed values (ref).

```r
colnames(dnds_bootmat)<-gene_list
dnds_bootmat<-as_data_frame(dnds_bootmat)

mdnds_bootmat<-melt(dnds_bootmat,variable.name="Gene",value.name="dn_ds")

## No id variables; using all as measure variables

#generate 95% confidence interval from bootstrap data
mdnds_summary<-mdnds_bootmat%>%
  group_by(Gene)%>%
  summarize(median=median(dn_ds,na.rm=T),
            lower=quantile(dn_ds,probs=0.025,na.rm=T),
            upper=quantile(dn_ds,probs=0.975,na.rm=T))

#calculate number of heteroplasmies observed in each gene
nhq_gene<-hq.spectrum%>%
  group_by(gene)%>%
  summarize(nhets=sum(nhets))
nhq_gene<-rbind(nhq_gene,
                data.frame(gene="ref",nhets=sum(hq.spectrum$nhets)))

#remove ND6 because no synonymous mutations are observed. This will give us an infinite
value for hn/hs

mdnds_summary2<-mdnds_summary%>%
  filter(Gene!="ND6")

mdmerged2<-mdmerged%>%
  filter(Gene!="ND6")

nhq_gene2<-nhq_gene%>%
  filter(gene!="ND6")

plt_hnhs<-ggplot(mdnds_summary2)+
  geom_point(aes(Gene,median))+
  geom_errorbar(aes(Gene,ymin=lower,ymax=upper))+
  theme_bw()+
  theme(axis.text.x=element_text(angle=90,
                                 size=14),
        axis.text.y=element_text(size=14),
        axis.title=element_text(size=16),
        text=element_text(size=14))+
  labs(x="Gene",y="hN/hS")+
  geom_point(data=mdmerged2,aes(Gene,hn_hs),color="red")+
  geom_text(data=nhq_gene2,aes(x=gene,y=10,label=nhets),angle=90,size=5)+
  geom_hline(yintercept = 1,linetype="dashed",color="blue") +
  ylim(c(0,11)) +
  ggtitle("Brain - mothers") +
        theme(plot.title = element_text(size=16, lineheight=.8, face="bold", hjust = 0.5))


plt_hnhs
```

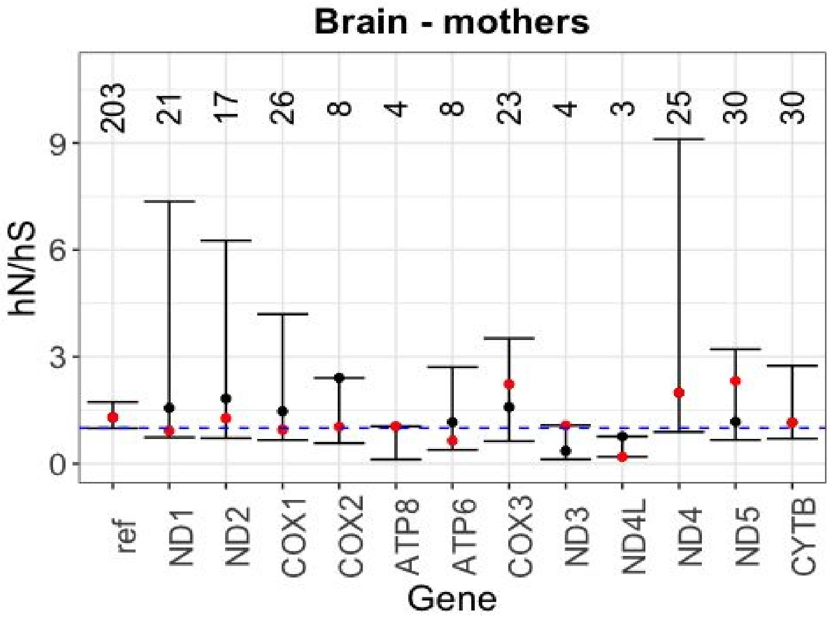


ND6 was excluded from plot because the observed value of hN/hS is infinite (no observed synonymous mutations in this gene, which makes it impossible to calculate hN/hS). *ref* indicates all protein-coding genes taken together.

## Results

The neutral distribution of hN/hS was generated by bootstrapping (100 replicates) from the observed mutation spectrum. For example, if there are 10 C>T mutations, 10 Cs are chosen uniformly at random and mutated to Ts, and hN/hS is calculated. 95% confidence intervals were calculated from the bootstrapped data.
When we look at the overall mutation spectrum for all protein-coding genes ('ref') for brain, muscle, and oocytes in mothers and pups, respectively, the observed hN/hS ratios (table below and red points in the figures below) is within the range of neutral expectations (blue dotted line and the black distribution). Thus, there is no evidence that purifying selection has impacted the distribution of observed mutations.

**hN/hS ratio calculated for the total protein-coding region ('ref') and separately for each individual gene for mutations observed in brain, muscle, and oocytes of mothers and pups, respectively.** hN is the number of nonsynonymous mutations per nonsynonymous sites; hS is the number of synonymous mutations per synonymous sites.

| | | Mothers | | | Pups | | |
|---|---|---|---|---|---|---|---|
| **Tissue** | **Gene** | **hN** | **hS** | **hN/hS** | **hN** | **hS** | **hN/hS** |
| brain | ATP6 | 0.01018 | 0.01579 | 0.64494 | 0.02648 | 0.03158 | 0.83843 |
| brain | ATP8 | 0.01982 | 0.01899 | 1.04405 | 0.02643 | 0.00000 | Inf |
| brain | COX1 | 0.01660 | 0.01749 | 0.94929 | 0.02359 | 0.02748 | 0.85845 |
| brain | COX2 | 0.01179 | 0.01143 | 1.03143 | 0.02750 | 0.01143 | 2.40668 |
| brain | COX3 | 0.03396 | 0.01523 | 2.22977 | 0.03396 | 0.01523 | 2.22977 |
| brain | CYTB | 0.02712 | 0.02349 | 1.15465 | 0.03302 | 0.02349 | 1.40566 |
| brain | ND1 | 0.02144 | 0.02332 | 0.91949 | 0.04002 | 0.01943 | 2.05965 |
| brain | ND2 | 0.01743 | 0.01370 | 1.27212 | 0.03351 | 0.02740 | 1.22319 |
| brain | ND3 | 0.01170 | 0.01091 | 1.07282 | 0.01560 | 0.02182 | 0.71521 |
| brain | ND4 | 0.02099 | 0.01054 | 1.99259 | 0.04199 | 0.01317 | 3.18814 |
| brain | ND4L | 0.00465 | 0.02439 | 0.19070 | 0.01395 | 0.00000 | Inf |
| brain | ND5 | 0.01935 | 0.00833 | 2.32143 | 0.02902 | 0.01458 | 1.98980 |
| brain | ND6 | 0.01014 | 0.00000 | Inf | 0.02282 | 0.01604 | 1.42265 |
| **brain** | **ref** | **0.01895** | **0.01457** | **1.30109** | **0.03052** | **0.01920** | **1.58918** |
| muscle | ATP6 | 0.02648 | 0.00000 | Inf | 0.02851 | 0.00526 | 5.41752 |
| muscle | ATP8 | 0.00000 | 0.03797 | 0.00000 | 0.02643 | 0.00000 | Inf |
| muscle | COX1 | 0.02009 | 0.01499 | 1.34066 | 0.02708 | 0.01749 | 1.54884 |
| muscle | COX2 | 0.01375 | 0.00000 | Inf | 0.02554 | 0.03429 | 0.74492 |
| muscle | COX3 | 0.03565 | 0.01015 | 3.51188 | 0.02886 | 0.01523 | 1.89530 |
| muscle | CYTB | 0.03184 | 0.01007 | 3.16274 | 0.02476 | 0.04027 | 0.61498 |
| muscle | ND1 | 0.02573 | 0.00777 | 3.31015 | 0.03859 | 0.03109 | 1.24131 |
| muscle | ND2 | 0.01609 | 0.01027 | 1.56568 | 0.02815 | 0.03082 | 0.91332 |
| muscle | ND3 | 0.03121 | 0.01091 | 2.86086 | 0.04291 | 0.02182 | 1.96684 |
| muscle | ND4 | 0.03199 | 0.01317 | 2.42906 | 0.02499 | 0.02897 | 0.86259 |
| muscle | ND4L | 0.01395 | 0.00000 | Inf | 0.05116 | 0.01220 | 4.19535 |
| muscle | ND5 | 0.02232 | 0.01250 | 1.78571 | 0.02455 | 0.01875 | 1.30952 |
| muscle | ND6 | 0.02790 | 0.03209 | 0.86940 | 0.02536 | 0.02406 | 1.05382 |
| **muscle** | **ref** | **0.02444** | **0.01126** | **2.17089** | **0.02837** | **0.02384** | **1.19016** |
| oocytes | ATP6 | 0.01426 | 0.01053 | 1.35438 | 0.01222 | 0.01053 | 1.16090 |
| oocytes | ATP8 | 0.01982 | 0.00000 | Inf | 0.00000 | 0.00000 | NA |
| oocytes | COX1 | 0.01398 | 0.01499 | 0.93263 | 0.01136 | 0.00500 | 2.27330 |
| oocytes | COX2 | 0.01375 | 0.01143 | 1.20334 | 0.01375 | 0.00000 | Inf |
| oocytes | COX3 | 0.01528 | 0.00508 | 3.01019 | 0.00679 | 0.00508 | 1.33786 |
| oocytes | CYTB | 0.02241 | 0.01007 | 2.22563 | 0.01179 | 0.01007 | 1.17138 |
| oocytes | ND1 | 0.02001 | 0.00777 | 2.57456 | 0.01429 | 0.01554 | 0.91949 |
| oocytes | ND2 | 0.01743 | 0.01370 | 1.27212 | 0.01072 | 0.01027 | 1.04379 |
| oocytes | ND3 | 0.01560 | 0.01091 | 1.43043 | 0.00390 | 0.00000 | Inf |
| oocytes | ND4 | 0.01300 | 0.01580 | 0.82234 | 0.00600 | 0.00263 | 2.27724 |
| oocytes | ND4L | 0.03256 | 0.03659 | 0.88992 | 0.00465 | 0.00000 | Inf |
| oocytes | ND5 | 0.01563 | 0.00625 | 2.50000 | 0.00893 | 0.00208 | 4.28571 |
| oocytes | ND6 | 0.01522 | 0.04011 | 0.37937 | 0.00507 | 0.00000 | Inf |
| **oocytes** | **ref** | **0.01657** | **0.01258** | **1.31702** | **0.00954** | **0.00563** | **1.69435** |

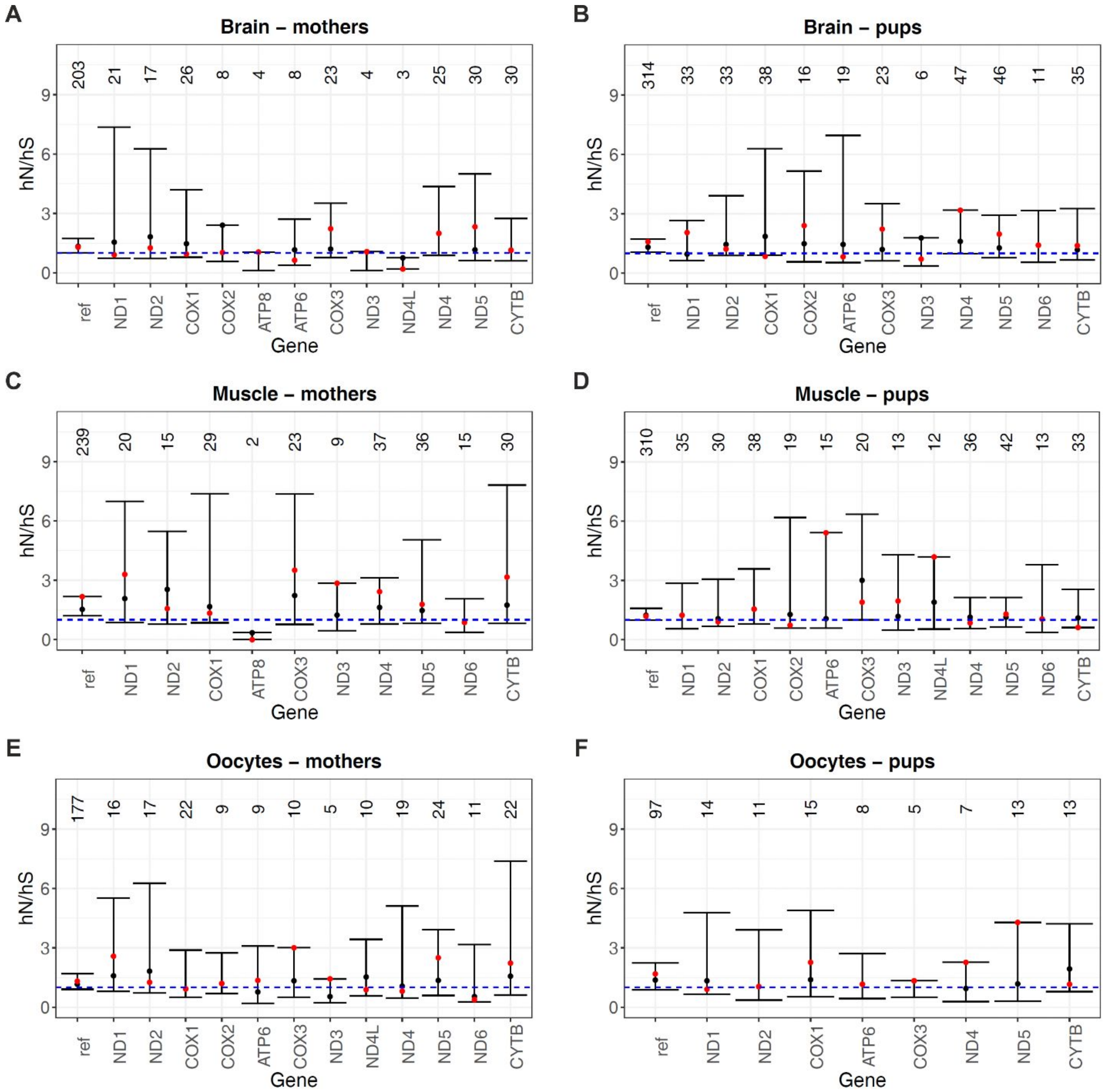


**hN/hS ratios calculated for the total protein-coding region ('ref') and separately for each individual gene.** Red points show the observed hN/hS ratio, black points and confidence intervals show the hN/hS ratio calculated under neutral expectation (bootstrapped for 100 replicates), and blue dotted line shows the hN/hS ratio of 1 (indicating absence of selection). **(A)** hN/hS ratio calculated for mutations observed in brain of mothers. ND6 was excluded from the plot because the observed value of hN/hS is infinite (no observed synonymous mutations in this gene, which makes it impossible to calculate hN/hS). **(B)** hN/hS ratio calculated for mutations observed in brain of pups. ATP8 and ND4L were excluded from the plot because no synonymous mutations were observed in these genes. **(C)** hN/hS ratio calculated for mutations observed in muscle of mothers. ATP6, COX2, and ND4L were excluded from the plot because no synonymous mutations were observed in these genes. **(D)** hN/hS ratio calculated for mutations observed in muscle of pups. ATP8 was excluded from the plot because no synonymous mutations were observed in this gene. **(E)** hN/hS ratio calculated for mutations observed in oocytes of mothers. ATP8 was excluded from the plot because no synonymous mutations were observed in this gene. **(F)** hN/hS ratio calculated for mutations observed in oocytes of pups. COX2, ND3, ND4L, and ND6 were excluded from the plot because no synonymous mutations were observed in these genes. ATP8 was excluded from the plot since no mutations (synonymous or nonsynonymous) were measured in this gene.
