# Supplementary material for: Age-related accumulation of de novo mitochondrial mutations in mammalian oocytes and somatic tissues
Source: PLoS Biol. 2020 Jul 15;18(7):e3000745. doi: 10.1371/journal.pbio.3000745 (PMC7363077; doi:10.1371/journal.pbio.3000745)

Note S3. Selection analysis - PhastCons scores

Code to test for selection using PhastCons scores (mutations in brain of mothers are used as an example)

phastCons analysis - mouse (brain - moms)

Arslan Zaidi, modified by Barbara Arbeithuber

1/14/2020

Introduction

Here, we are interested in looking for signatures of selection in mouse mtDNA by studying the distribution of mutations occurring in the mouse mtDNA. We have already shown that hN/hS values for protein-coding genes are within the neutral distribution. Thus, there appears to be no evidence for selection acting against mutations occurring in these regions. To more fully look at selection acting on all mutations (including those occurring in non-coding regions), we will now investigate whether mutations are less likely to occur in conserved regions.

Methodology

To do this, I downloaded phastCons (PhastCons60wayEuarchontoGlires table) scores from the UCSC genome browser for the mouse reference mtDNA genome. To this, I added the table of mtDNA mutations observed using duplex sequencing.

```
##
## Attaching package: 'dplyr'
## The following objects are masked from 'package:stats':
##
##     filter, lag
## The following objects are masked from 'package:base':
##
##     intersect, setdiff, setequal, union
##
## Attaching package: 'data.table'
## The following objects are masked from 'package:dplyr':
##
##     between, first, last
## here() starts at /Users/babsi/duplex_analysis/2019_mm_pedigrees
#phastcons scores
pcons<-fread(("2019-01_analysis/tables/mm9_eur_phastcons.txt"),header=F)
colnames(pcons)<-c("position","phastcons")
#duplex sequencing mutations
dat<-fread(("2019-01_analysis/tables/Br_mom_full.txt"),header=T)
#heteroplasmies only
hq<-dat%>%
  filter(minor!=".")

hq2=hq%>%
  distinct(position,major,minor)

pcons2=merge(pcons,hq,by="position",all.x=T)
```

Annotate each position as being either ‘heteroplasmic’ or not and either ‘conserved’ or not. Conserved sites are those that have a phastcons score of greater than 0.9 and Not-conserved sites are those that have a

phastcons score of less than or equal to 0.1. Plot the distribution of phastcons scores and the median for both heteroplasmic and homoplasmic sites.

```
options(repr.plot.width=3.5, repr.plot.height=3)
pcons2=pcons2%>%
  mutate(heteroplasmy=case_when(is.na(major)=="TRUE"~"n",
          TRUE~"h"),

     conserved=case_when(phastcons>0.9~"conserved",
          phastcons<0.1~"not conserved"))

pcons2.sum=pcons2%>%
  group_by(heteroplasmy)%>%
  summarize(lower=quantile(phastcons,probs=0.025),
       upper=quantile(phastcons,probs=0.975),
       median=quantile(phastcons,probs=0.5))


pcons3=pcons2%>%
  filter(is.na(conserved)=="FALSE")

p= ggplot(pcons3)+
  geom_point(position="jitter",
       aes(heteroplasmy,phastcons,color=conserved),size=1, alpha=0.9)+
  geom_point(data=pcons2.sum,aes(heteroplasmy,median),color="black")+
  theme_bw()+
  ggtitle("Brain - Mothers") +
  theme(plot.title = element_text(size=10, lineheight=.8, hjust = 0.5, face="bold")) +
  guides(color = guide_legend(title="")) +
  theme(legend.position="bottom") +
  labs(x="Mutation status",
  y="PhastCons score")

#ggsave(filename="2020-01-14_PhastCons_Br_mom.pdf", plot=p, , width = 3.5, height = 3)
p
```

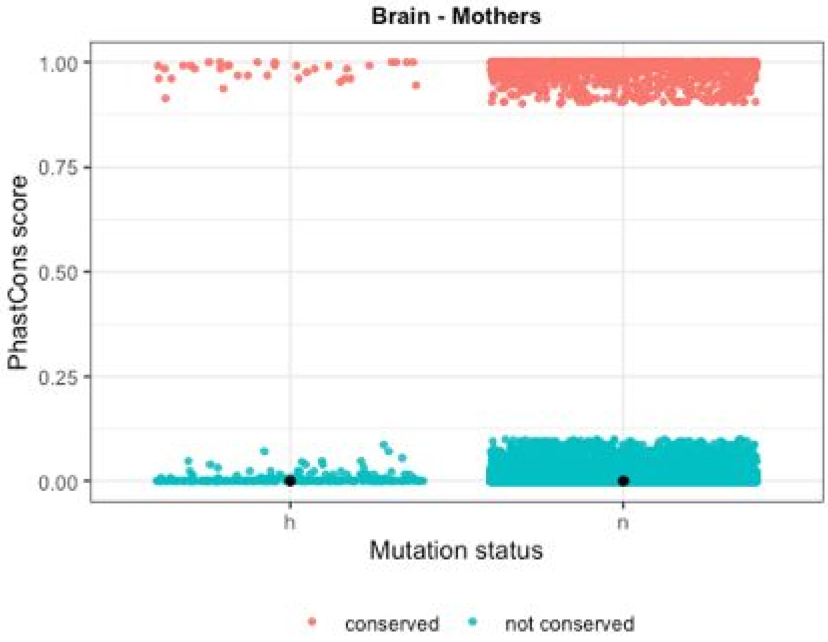

It doesn't look like non-heteroplasmic sites have a higher average phastcons score compared to heteroplasmic sites. Let's investigate this more formally using a Fisher's Exact test. To do this, make a 2x2 contingency table.

```
cont.table=pcons3%>%
  group_by(conserved,heteroplasmy)%>%
  summarize(n=length(phastcons))%>%
  dcast(conserved~heteroplasmy,value.var="n")

cont.table=cont.table[,-1]
rownames(cont.table)=c("conserved","not_conserved")
colnames(cont.table)=c("h","n")


fisher.test(cont.table,alternative = "l")
##
##  Fisher's Exact Test for Count Data
##
## data:  cont.table
## p-value = 0.9551
## alternative hypothesis: true odds ratio is less than 1
## 95 percent confidence interval:
##  0.00000 1.83551
## sample estimates:
## odds ratio
##   1.348022
```

Fisher's exact test shows that mutations aren't any more likely to occur at non-conserved sites than at conserved sites. Let's test this non-parametrically using a permutation test. To do this, I will 'rotate' the mtDNA genome by a random number and test the association between conservation status and mutation status. I will calculate the Odd's ratio (odds of observing a mutation at non-conserved site/odds of observing a mutation at

a conserved site). We expect this number to be significantly greater than 1 in the observed data compared to permuted data.

```
OR=(cont.table[2,1]/sum(cont.table[2,]))/
  (cont.table[1,1]/sum(cont.table[1,]))


OR
## [1] 0.7467047
```

Turns out, the Odd's ratio is actually less than 1, which means that mutations are more likely to occur at conserved sites rather than non-conserved sites. Let's calculate a one-sided p-value for this to see just how significant this result is.

```
#function to rotate the mtDNA and calculate Odd's ratio
frotate=function(){

  step=sample(16297,1)
  ppcons=pcons2$position+step
  ppcons[which(ppcons>16296)]=ppcons[which(ppcons>16296)]-16296
  ppcons.df=cbind(pcons2[ppcons,c(1:5)],pcons2[,6])
  colnames(ppcons.df)[6]="conserved"
  pcont.table=ppcons.df%>%
    filter(is.na(conserved)=="FALSE")%>%
    group_by(conserved,heteroplasmy)%>%
    summarize(n=length(phastcons))%>%
    dcast(conserved~heteroplasmy,value.var="n")

  pcont.table=pcont.table[,-1]
  OR=(pcont.table[2,1]/sum(pcont.table[2,]))/
    (pcont.table[1,1]/sum(pcont.table[1,]))
  return(OR)
}

ormat=matrix(NA,10000,ncol=2)
#pb=txtProgressBar(min=0,max=10000,style=3)
for(i in 1:1e4){
  ormat[i,1]=i
  ormat[i,2]=frotate()
#setTxtProgressBar(pb,i)
}

print(length(which(ormat[,2]<OR))/length(ormat[,2]))
## [1] 0.1168
options(repr.plot.width=4, repr.plot.height=3)

ormat=as.data.frame(ormat)
colnames(ormat)=c("replicate","OR")

pval=length(which(ormat[,2]<OR))/length(ormat[,2])

q= ggplot()+
  geom_histogram(data=ormat,aes(OR),
```

```
      color="black",
      fill="white")+
theme_bw()+
geom_vline(xintercept=OR,color="red")+
theme_bw()+
theme(panel.grid = element_blank())+
annotate(geom="text",
  x=OR,
  y=1500,
  label=paste("Pvalue:",round(pval,2)))+
labs(x="Odd's ratio",
  y="Count")

#ggsave(filename="2020-01-14_Hist_Br_mom.pdf", plot=q, , width = 4, height = 3)
q
## `stat_bin()` using `bins = 30`. Pick better value with `binwidth`.
```

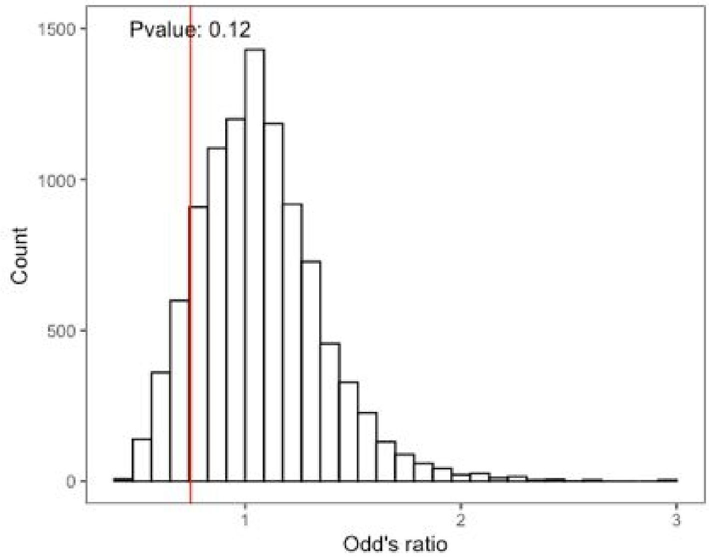

So, even though the odd's ratio is less than 1, it is not significantly different. Thus, there is no strong evidence for or against negative selection acting against mutations occuring in the mouse mtDNA.

<u>Results</u>

PhastCons scores were not significantly lower for mutated sites relative to sites that were not mutated (with the exception of brain tissue in pups). Thus, there is little evidence to support the role of negative selection in shaping the observed distribution of mutations in mouse mtDNA in the tissues and age groups analyzed.

PhastCons scores of mutated sites (h) and sites that were not mutated (n). No obvious deviation in the distribution of conserved and not conserved PhastCons scores between mutated and not mutated sites can be detected. Further statistical analysis was performed: Fisher's Exact test p= 0.955, 0.993, 0.747, 0.988, 0.474, and 0.560 for brain in mothers, muscle in mothers, oocytes in mothers, brain in pups, muscle in pups, and oocytes in pups.

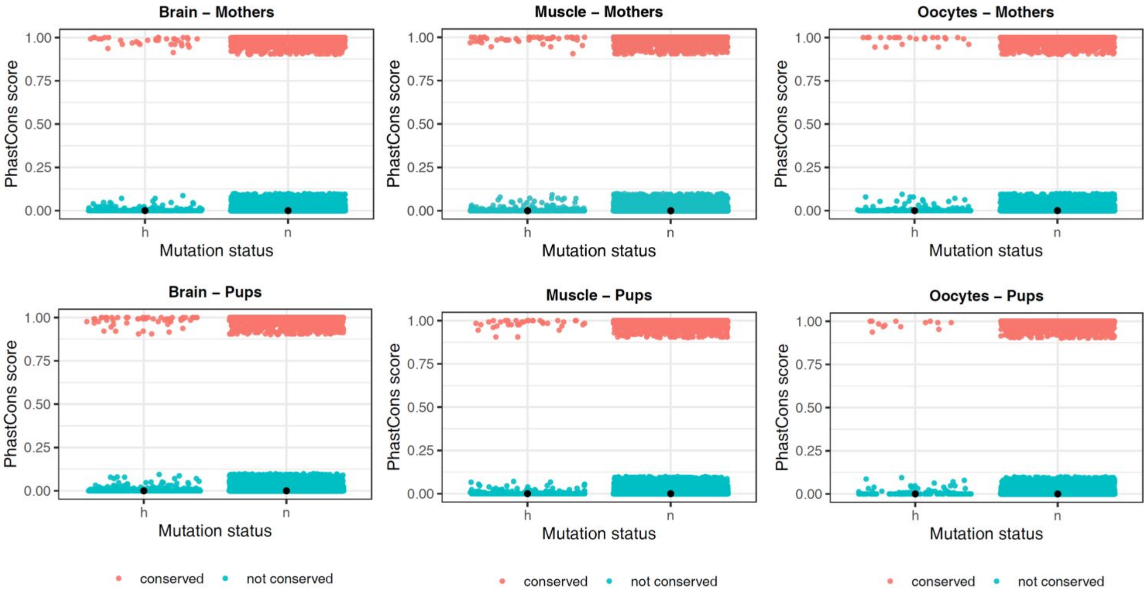

Association between conservation status and mutation status tested non-parametrically. The mtDNA genome was ‘rotated’ by a random number and the association between conservation status and mutation status was tested non-parametrically (permutation test). The Odd’s ratio (odds of observing a mutation at non-conserved site/odds of observing a mutation at a conserved site) was calculated (red line). A significant deviation is only observed in brain of pups.

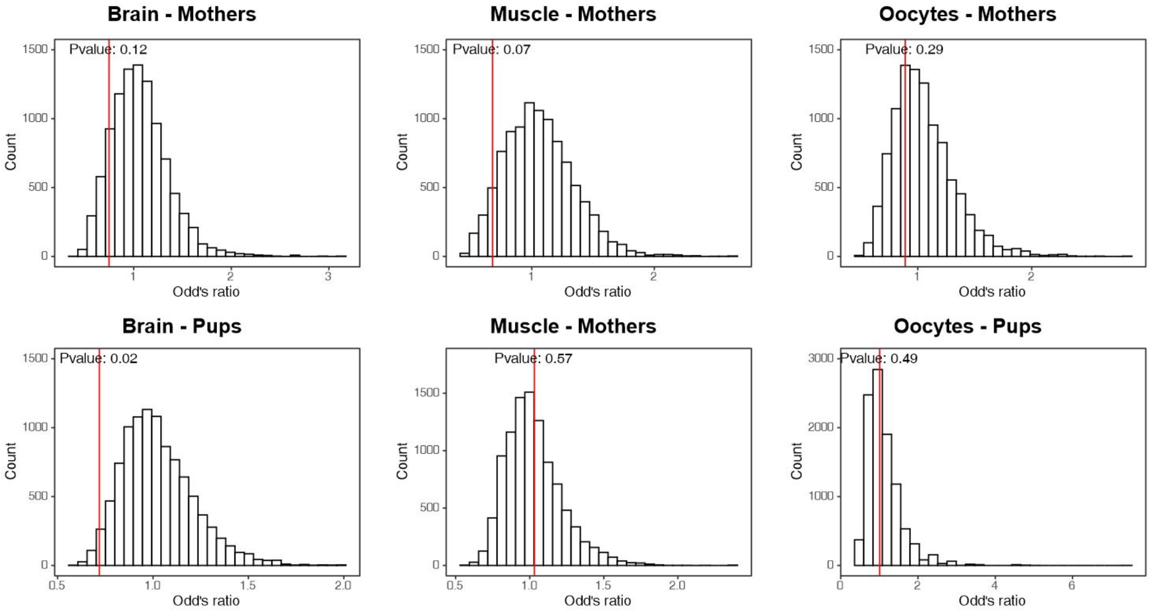

Supplement: S3 Note — (PDF) [file pbio.3000745.s042.pdf]
