# Supplementary material for: Age-related accumulation of de novo mitochondrial mutations in mammalian oocytes and somatic tissues
Source: PLoS Biol. 2020 Jul 15;18(7):e3000745. doi: 10.1371/journal.pbio.3000745 (PMC7363077; doi:10.1371/journal.pbio.3000745)
Supplement: S4 Note — (PDF) [file pbio.3000745.s043.pdf]

Note S4. Mutations in regulatory D-loop regions

Code to test for enrichment for mutations in regulatory regions of the D-loop (mutations in brain of mothers are used as an example)

Test of enrichment for mutations in regulatory regions -mouse (brain - moms)

Arslan Zaidi, modified by Barbara Arbeithuber
2/14/2020

Introduction

We would like to test whether there is a depletion of observed mutations in regulatory regions of the D-loop compared to the non-regulatory region of the D-loop. The idea is that because of the known functional importance of regulatory regions, fewer mutations might be observed in these regions due to purifying selection. However, if there is no depletion, it does not mean selection does not operate on these regions, just that there hasn't been enough time for selection to remove these mutations or that we are observing the true mutation spectrum without the action of selection.

Methodology

To test this, I downloaded start and stop positions for 5 regulatory regions in mice (ETAS1, ETAS2, CSB1, CSB2, and CSB3) from the mouse mtDNA genbank file (accession number: NC_005089.1). Then, I tested using either the Chi-squared test or Fisher's exact test whether there was an enrichment of mutations in regulatory regions relative to non-regulatory regions of the D-loop.

```
##
## Attaching package: 'dplyr'
## The following objects are masked from 'package:stats':
##
##     filter, lag
## The following objects are masked from 'package:base':
##
##     intersect, setdiff, setequal, union
##
## Attaching package: 'data.table'
## The following objects are masked from 'package:dplyr':
##
##     between, first, last
## here() starts at /Users/babsi/duplex_analysis/2019_mm_pedigrees
#duplex sequencing mutations
dat<-fread(("2019-01_analysis/tables/Br_mom_full.txt"),header=T)
```

Specify start and stop positions for the regulatory and non-regulatory regions of the D-loop.

```
reg_regions=data.table(
region=rep("Regulatory",5),
name=c("ETAS1","ETAS2","CSB1","CSB2","CSB3"),
start=c(15451,15515,16035,16089,16114),
stop=c(15509,15558,16058,16104,16131)
)
d_regions=data.table(
region=rep("Dloop",6),
name=rep("Dloop",6),
start=c(15423,15510,15559,16059,16105,16132),
stop=c(15450,15514,16034,16088,16113,16299)
)
dloop=rbind(d_regions,reg_regions)
```

Calculate the length of each region. This will be important later to generate expected numbers of mutations.

```
dloop=dloop%>%
```

```
mutate(length=stop-start)
dloop.sum=dloop%>%
group_by(region)%>%
summarize(length=sum(length))
dloop.sum
## # A tibble: 2 x 2
##   region     length
##   <chr>       <dbl>
## 1 Dloop         710
## 2 Regulatory    156
```

Annotate heteroplasmies with which region they belong in and then calculate the observed number of heteroplasmies within each region.

```
dat$name=NA
dat$region=NA
for(i in 1:nrow(dloop)){
ix=which(dat$position > dloop$start[i] & dat$position < dloop$stop[i])
dat$region[ix]=dloop$region[i]
dat$name[ix]=dloop$name[i]
}
dat.sum=dat%>%
group_by(region)%>%
summarize(n=length(which(minor!=".")))
dat.sum
## # A tibble: 3 x 2
##   region         n
##   <chr>      <int>
## 1 Dloop         47
## 2 Regulatory     7
## 3 <NA>         271
```

Calculate the expected number of heteroplasmies based on the length of each region.

```
dloop.sum=merge(dloop.sum,dat.sum)
dloop.sum=dloop.sum%>%
mutate(exp.n=length/sum(length)*sum(n))
dloop.sum
##       region length  n     exp.n
## 1      Dloop    710 47 44.272517
## 2 Regulatory    156  7  9.727483
```

Perform the Chi-squared test of independence and Fisher's exact test to determine if there is a non-random distribution of heteroplasmies between regulatory and non-regulatory regions.

```
chisq.test(dloop.sum[,c(3,4)])
##
##  Pearson's Chi-squared test with Yates' continuity correction
##
## data:  dloop.sum[, c(3, 4)]
## X-squared = 0.2111, df = 1, p-value = 0.6459
fisher.test(dloop.sum[,c(3,4)])
## Warning in fisher.test(dloop.sum[, c(3, 4)]): 'x' has been rounded to
## integer: Mean relative difference: 0.01009323
##
##  Fisher's Exact Test for Count Data
##
## data:  dloop.sum[, c(3, 4)]
## p-value = 0.5983
## alternative hypothesis: true odds ratio is not equal to 1
## 95 percent confidence interval:
##  0.4740201 5.1502049
```

```
## sample estimates:
## odds ratio
##   1.520016
```

The non-significant P-values tell us that the distribution of mutations appears to be random with respect to the functional annotation within the D-loop. This result is consistent with our other tests showing that there appears to be no evidence of purifying selection on this set of mutations. Therefore, the distribution of mutations observed offers a snapshot of the mutation spectrum without the effects of selection.

Results:

We did not observe a depletion of mutations in regulatory regions of the D-loop (ETAS1, ETAS2, CSB1, CSB2, and CSB3) compared to the non-regulatory region of the D-loop. Both tests, the Chi-squared test or Fisher's exact test did not show significant enrichment of mutations in regulatory regions relative to non-regulatory regions of the D-loop in any of the tissues and age groups. Though, despite that we did not observe a depletion, it does not mean selection does not operate on these regions, it just means that there has not been enough time for selection to remove these mutations or that we are observing the true mutation spectrum without the action of selection.

	**p-value (Chi-squared test)**	**p-value (Fisher's Exact test)**
**Brain - mother**	0.6459	0.5983
**Brain - pup**	0.7444	0.7792
**Muscle - mother**	0.9469	1
**Muscle - pup**	0.1624	0.1406
**Oocytes - mother**	0.4335	0.4841
**Oocytes - pup**	0.9535	1
