# Supplementary material for: Age-related accumulation of de novo mitochondrial mutations in mammalian oocytes and somatic tissues
Source: PLoS Biol. 2020 Jul 15;18(7):e3000745. doi: 10.1371/journal.pbio.3000745 (PMC7363077; doi:10.1371/journal.pbio.3000745)
Supplement: Attachment — Submitted filename: Editorial comments.pdf [file pbio.3000745.s045.pdf]

May 15, 2020

Dear Editor,

We are glad that the reviewers did not have any further concerns.
Below, we address the comments regarding the ethics statement and data policy, as well as regarding the formatting requirements. Our replies are shown in blue.

Thank you for handling our manuscript.

Sincerely,

Kateryna Makova
Corresponding author

ETHICS STATEMENT:

-- Please include the full name of the IACUC/ethics committee that reviewed and approved the animal care and use protocol/permit/project license. Please also include an approval number (I cannot currently see this).

Committee: Institutional Animal Care and Use Committee (Protocol #44472). This information was now added to the Methods section.

-- Please include the specific national or international regulations/guidelines to which your animal care and use protocol adhered. Please note that institutional or accreditation organization guidelines (such as AAALAC) do not meet this requirement.

Federal guidelines followed: Animal Welfare Act (Public Law 99-198). This information was now added to the Methods section.

-- Please include information about the form of consent (written/oral) given for research involving human participants. All research involving human participants must have been approved by the authors' Institutional Review Board (IRB) or an equivalent committee, and all clinical investigation must have been conducted according to the principles expressed in the Declaration of Helsinki.

We did not include research on any human subjects in this work.

DATA POLICY:

You may be aware of the PLOS Data Policy, which requires that all data be made available without restriction: http://journals.plos.org/plosbiology/s/data-availability. For more information, please also see this editorial: http://dx.doi.org/10.1371/journal.pbio.1001797

Many thanks for depositing your raw data in the SRA; however, we also need the numerical values that underlie the data summarized in the figures and results of your paper be made available in one of the following forms:

1) Supplementary files (e.g., excel). Please ensure that all data files are uploaded as 'Supporting Information' and are invariably referred to (in the manuscript, figure legends, and the Description field when uploading your files) using the following format verbatim: S1 Data, S2 Data, etc. Multiple panels of a single or even several

figures can be included as multiple sheets in one excel file that is saved using exactly the following convention: S1_Data.xlsx (using an underscore).

2) Deposition in a publicly available repository. Please also provide the accession code or a reviewer link so that we may view your data before publication.

Regardless of the method selected, please ensure that you provide the individual numerical values that underlie the summary data displayed in the following figure panels as they are essential for readers to assess your analysis and to reproduce it: Figs 2-5 and most of the Supplementary Figures. NOTE: the numerical data provided should include all replicates AND the way in which the plotted mean and errors were derived (it should not present only the mean/average values).

Please also ensure that figure legends in your manuscript include information on where the underlying data can be found, and ensure your supplemental data file/s has a legend.

While most of the data was already included in Supplementary tables, we now also deposited the data relevant for plotting the individual Figures and Supplementary Figures to GitHub (https://github.com/makovalab-psu/mouse-duplexSeq). Information on the availability of this data was added to the corresponding figure legends.

Please ensure that your Data Statement in the submission system accurately describes where your data can be found.

Confirmed.

BLOT AND GEL REPORTING REQUIREMENTS:

For manuscripts submitted on or after 1st July 2019, we require the original, uncropped and minimally adjusted images supporting all blot and gel results reported in an article's figures or Supporting Information files. We will require these files before a manuscript can be accepted so please prepare and upload them now. Please carefully read our guidelines for how to prepare and upload this data: https://journals.plos.org/plosbiology/s/figures#loc-blot-and-gel-reporting-requirements

No blots or gels were included.

PLOS Formatting Requests

For: “Age-related accumulation of de novo mitochondrial mutations in mammalian oocytes and somatic tissues”

METADATA CHECK

Please ensure that all information entered in the submission form is correct. This information will be indexed in PubMed and published by *PLOS Biology* as written.

¨ An **Early Version** of your manuscript:

o WILL be published online ahead of the final version

If you (or your institution) are planning a press release for this article, please contact us as soon as possible to ensure that an early version of your paper is not published.

We are planning a press release for this article, so please do not publish an early version of this article.

¨ Please check the **authorship and affiliations** list on the title page of your manuscript. These details will be used to index the authorship of your manuscript and therefore should be checked carefully for any errors.

The authorship and affiliations list on the title page are correct.

¨ Please ensure that if you have any protocols associated with protocols.io in your manuscript, they are made public before publication to guarantee immediate access to the methodological details.

We did not submit any protocols to protocols.io.

Please confirm that the following elements are correct and written in full sentences. Please update as needed.

¨ **Financial Disclosure**

¨ **Competing Interests**

¨ **Data Availability Statement**

All these elements are correct and written in full sentences.

MANUSCRIPT REQUIREMENTS

Please update your manuscript with the following changes:

1. Please provide a detailed Financial Disclosure statement in the submission form. Include grant numbers and URLs as appropriate, as well as the initials of the authors who received the funding. This is published with the article, therefore it should be completed in full sentences and contain the exact wording you wish to be published.

Please add the statement 'The funders had no role in study design, data collection and analysis, decision to publish, or preparation of the manuscript' to the end of your Financial Disclosure statement on the submission form. If this statement is inaccurate, please describe the role the funders had.

The statement was added to the end of our Financial Disclosure statement, and the statement was moved from the manuscript to the submission form.

If you received no funding, please add the statement 'The author(s) received no specific funding for this work' to your submission form.

N/A

2. In your Data Availability Statement please ensure you have provided all necessary access information in complete sentences. This statement will appear on the published version of your manuscript.

All necessary access information is provided.

3. Please ensure your short title is correct in the submission form, including symbols, italicisation etc. Your short title will be typeset from the submission form directly.

The short title in the submission form is correct.

4. Please indicate the corresponding author on the title page with an asterisk (*) and include the corresponding author's email address. Only an asterisk and the email address itself are required.

The corresponding author on the title page is indicated with an asterisk and an email address is included.

5. Please ensure that all authors have at least one affiliation listed on the title page and that all listed affiliations are attributed to at least one author. Affiliations should be listed in ascending numeric order in the affiliation list and should match the information provided in the Authors information in the submission form. Affiliations should include an institution, a city, and a country (no postcode). US affiliations need the state as well, written out in full (with no abbreviations).

The affiliations were checked and postcodes were removed.

6. Please ensure that all author names are spelled correctly in the manuscript file and the submission form, including the use of a middle name/initials, and any special characters (accents, umlauts, etc.). Please place first/given names before last/family names. We noticed that Marzia A Cremona and Francisco J Diaz are spelled differently and ask that you ensure they match verbatim. Please be aware that correct spelling and formatting of author names in the article file is vital for accurate indexing in PubMed.

We checked the spelling of those names and corrected the errors.

7. Please remove the funding information from your manuscript file. The published version is pulled automatically from the Financial Disclosure section of the submission form.

The funding information was removed from the manuscript file.

8. Please remove the Competing Interests section from your manuscript file. The published version is pulled automatically from the Competing Interests section of the submission form.

The Competing Interests section was removed from the manuscript file.

9. Please remove your Author Contribution section from your manuscript file as this information is automatically pulled from the Authors information in the submission form.

The Author Contribution section was removed from the manuscript file.

10. Please remove your Data Statement section from your manuscript file as this information is automatically pulled from the Data Availability question on the submission form.

The Data Statement section was removed from the manuscript file.

11. Please ensure that your Ethics Statement is available at the beginning of your Methods section, under a subheading 'Ethics Statement' in its entirety. Please see our guidance for Human Subjects Research here: https://journals.plos.org/plosbiology/s/human-subjects-research and for Animal Research here: https://journals.plos.org/plosbiology/s/animal-research

We added the ethics statement to the beginning of the Methods section.

12. Your references need to be formatted according to the ICMJE standard reference style. PLOS uses the numbered citation [citation-sequence] method and first six authors, et al. Authors should be listed as "Last name, First Initial". Please note that references should be cited in square [ ] brackets and not round ( ) brackets to ensure that they are correctly typeset. Additionally, the reference list should be formatted as "1." and not "[1]". Please adhere to the PLOS style for all references. For a full outline see our Author Guidelines: http://journals.plos.org/plosbiology/s/submission-guidelines#loc-references

All references were adjusted to this format.

13. Please put all reference citations in square brackets, with no spaces, separated by either commas or dashes, e.g. [1,2,3] or [1-3].

All reference citations were adjusted to this format.

14. Please make sure all references are cited in ascending numerical order in the text.

All references are cited in ascending numerical order in the text.

15. Please ensure that all supporting information file names use the following format verbatim in the file inventory. The file names themselves must use an underscore rather than a space, i.e. S1_Fig.tif, S1_Table.xlsx, S1_Text.doc, S1_Data.xlsx, S1_PRISMA_Checklist.docx, etc. Each file type should be numbered starting at 1. Please also make sure that the file description matches the file name (e.g. S1_Fig.tif is listed as S1 Fig).

The mentioned format verbatim was used in the supporting file inventory.

16. Please ensure that each supporting information file has a legend at the end of the manuscript file, after the Reference list.

Each supporting information file has a legend at the end of the manuscript file.

17. Please ensure that all supporting information legend titles use the following format verbatim in all in-text citations (including spelling and capitalization): S1 Fig, S1 Table, S1 Text, S1 Data, S1A Fig etc. Each file type should be numbered starting at 1.

The mentioned format verbatim was used in all supporting information legend titles and in-text citations.

18. Please note that your submission cannot contain any references to generic "supporting information" files and instead must refer to specific files using our naming convention (S1 Fig, S1 Table, S1 Text, S1 Data, etc.)

Only references to specific supporting files are used.

If Supporting Information files are included with your article, note that these are not copyedited and will be published as they are submitted. Please ensure that these files are legible and of high quality (at least 300 dpi) in an easily accessible file format. For this reason, please be aware that any references listed in an SI file will not be indexed. For more information, see our Supporting Information guidelines.

All Supporting Information files are legible and of high quality.

FIGURE REQUIREMENTS

PLOS offers a figure-checking tool, PACE (http://pacev2.apexcovantage.com/), to help authors to ensure all figures meet PLOS requirements so that the quality of published figures is as high as possible. **Please use this tool to help you address the requests below.**

PACE is a digital diagnostic and conversion tool for figure files. It will provide information about any failed check(s) and, if able, will automatically convert the figure file into an acceptable file that passes quality checks. PACE requires you to register for an account to ensure your figure files are processed securely.

Please complete the following instructions regarding your figure files:

1. Please ensure that all main figure files follow PLOS naming conventions in the File Inventory. The file name should be in the format "Fig1.tif" and the file description should be "Fig 1".

All main figure files follow PLOS naming conventions in the File Inventory.

2. Please ensure that all main figure files are cited in ascending numerical order in the main text of the article.

All main figure files are cited in ascending numerical order in the main text.

3. Please ensure that all main figures are referenced in the manuscript as Fig 1, Fig 2, etc. (including capitalization), rather than Figure 1, Figure 2, etc. Please note, however, that the file names themselves must not include the space, i.e. Fig1.tif, Fig2.eps.

Please use the following examples of naming conventions for citing multiple figures in your manuscript:

- Figs 3 and 4

- Figs 3, 4, and 7

- Figs 1-5

- Fig 3A and 3B

- Figs 3A and 7B

All main figures are referenced in the manuscript as Fig 1, Fig 2, etc. using those naming conventions for citing multiple figures.

4. Place each main figure legend directly below the paragraph in which that figure is first referenced.

Each main figure legend is placed directly below the paragraph in which that figure is first referenced.

5. Please note that all figures and supporting information files will be published under the Creative Commons Attribution License (creativecommons.org/licenses/by/4.0/). Under this license, authors retain ownership of the copyright for their article, but allow anyone to download, reuse, reprint, modify, distribute, and/or copy articles in PLOS journals for any reason, even commercially, so long as the original

authors and source are cited. Permission is not required from PLOS or authors to reuse any content as long as proper attribution is provided.

We ask that you please confirm via email to plosbiology@plos.org that you either created the following figure panels or have written permission from the copyright holder to publish them under a CC-BY license:

- Arrows in Fig S1 → We now removed panel A of Fig S1 to avoid any copyright issues.

- Galaxy Project logo (this will need to be removed) → We removed the logo from the figure.

- Mouse, brain and test tube graphics in Fig S2 → We did draw those elements by ourselves.

- Image of machinery in Fig S2→ We removed the image of the MiSeq machinery from the plot.

Please note that any written confirmation you send to us must clarify that the copyright holder understands and agrees to the terms of this license. Note that an email confirmation is acceptable.

Please note that this is your only chance to adjust figures, changes to figures (including resizing) at proof will not be accepted.

For a full outline, please see our figure and table preparation guidelines.

We now follow all of the mentioned instructions regarding our figure files and checked them using PACE.

You can also upload a Striking Image to accompany your article if one is available. If this image is judged to be suitable, it may be featured on our website. Images should be high resolution, eye-catching, single panel images. If your image is from someone other than yourself, please ensure that the artist has read and agreed to the terms and conditions of the Creative Commons Attribution License. Note: we cannot publish copyrighted images.

A cover image was uploaded to accompany our article. The legend to the cover art: "Mouse cumulus oocyte complex (COC) isolated from an antral follicle of a CD-1 mouse. Image taken by Francisco Diaz using Olympus Te2000 microscope with 40x magnification."
